# Antitumor and Antimicrobial Activity of Some Cyclic Tetrapeptides and Tripeptides Derived from Marine Bacteria

**DOI:** 10.3390/md13053029

**Published:** 2015-05-15

**Authors:** Subrata Chakraborty, Dar-Fu Tai, Yi-Chun Lin, Tzyy-Wen Chiou

**Affiliations:** 1 Department of Chemistry, National Dong Hwa University, Hualien 974, Taiwan; E-Mail: cpapu2000@yahoo.com; 2 Department of Life Science, National Dong Hwa University, Hualien 974, Taiwan; E-Mails: a30483@yahoo.com.tw (Y.C.L.); twchiou@mail.ndhu.edu.tw (T.W.C.)

**Keywords:** antibiotic, antitumor, cyclic tetrapeptide, cyclic tripeptide

## Abstract

Marine derived *cyclo*(Gly-l-Ser-l-Pro-l-Glu) was selected as a lead to evaluate antitumor-antibiotic activity. Histidine was chosen to replace the serine residue to form *cyclo*(Gly-l-His-l-Pro-l-Glu). Cyclic tetrapeptides (CtetPs) were then synthesized using a solution phase method, and subjected to antitumor and antibiotic assays. The benzyl group protected CtetPs derivatives, showed better activity against antibiotic-resistant *Staphylococcus aureus* in the range of 60–120 μM. Benzyl group protected CtetPs 3 and 4, exhibited antitumor activity against several cell lines at a concentration of 80–108 μM. However, shortening the size of the ring to the cyclic tripeptide (CtriP) scaffold, c*yclo*(Gly-l-Ser-l-Pro), *cyclo*(Ser-l-Pro-l-Glu) and their analogues showed no antibiotic or antitumor activity. This phenomenon can be explained from their backbone structures.

## 1. Introduction

The discovery and study of antimicrobial peptides (AMPs) has greatly affected the field of drug design [1,2]. Many of these host-defense peptides (HDPs) are also anticancer peptides (ACPs) with potent abilities to inhibit the growth of a wide range of cancer cells [3,4,5,6]. Although the antimicrobial activity of peptides can now be predicted [7,8], the potency of these antimicrobial peptides is normally not as strong as certain conventional antibiotics. Their major strengths are broad-spectrum abilities to kill multi-drug-resistant bacteria at similar concentrations, rapid bacterial activity, and low propensity for resistance [9,10].

Designing and creating orally potent constrained peptides with cell-penetrating and targeted protein interacting properties is an emerging area of drug discovery. To increase the efficacy of this kind of peptide, with cyclization, it is possible to systematically improve both the pharmacodynamics and pharmacokinetic properties [11,12,13]. Cell-permeable bicyclic peptides are known to gain inhibition properties [14,15]. Another strategy is to use a small, low molecular weight peptide [16,17] which lowers the minimum inhibitory concentration (MIC) as well as the cost of synthesis. Their sequence can also be altered to increase specificity and activity; or to decrease toxicity [18]. Cyclic tetrapeptides (CtetPs) are constrained by privileged secondary structures, can self-assemble, and stack up to form hollow, β-sheet-like nanotubes [19]. They have been characterized as potent and highly selective molecules in a diverse range of therapeutic areas such as the antiproliferative agent *cyclo*(l-Pro-l-Leu-l-Pro-l-Leu) [20], tyrosinase inhibitor *cyclo*(l-Pro-l-Tyr-l-Pro-l-Val) [21], and many histone deacetylase inhibitors [22,23,24]. The discovery of these bioactive peptides has led to further development of their antagonists or agonists. Numerous efforts have been focused on building a variety of peptidomimetics [25,26,27]. Recently, a new antibiotic teixobactin [28] was found to inhibit cell wall synthesis. This linear peptide linked cyclic pseudopeptide is effective against drug-resistant pathogens in a number of animal models of infection. Antibiotics of this type are likely to avoid development of resistance. These types of pseudopeptides use the conformational preferences of fused rings, stereochemistry, and strong covalent bonds to define their shape for molecular recognition [29]. Thus, rigid peptidomimetics are useful tools to construct confined nanospaces [30] for structure-activity studies in peptide-based drug discovery [8].

Antimicrobial activity of *cyclo*(Gly-l-Ser-l-Pro-l-Glu) **1** against *Bacillus subtilis* has been reported [31]. This anionic AMP was first isolated from the *Ruegeria* strain of marine bacteria and found to possess moderate bactericidal activity. Its structure was elucidated based on 1D and 2D NMR data, followed by determination of absolute configuration [32,33]. Recently, the coordination of *cyclo*(Gly-l-Ser-l-Pro-l-Glu) with a lead ion was reported [19]. However, their antitumor activity has never been explored and their antibiotic activity against other bacteria has not been reported. Herein, several analogs based on the target *cyclo*(Gly-l-Ser-l-Pro-l-Glu) were synthesized. Their antibiotic and antitumor activities were then evaluated with minimal inhibitory concentrations (MICs) assay [34] and MTT assay [35].

## 2. Results and Discussion

The vast majority of ACPs are cationic and adopt a molecular architecture such as α-helical(α-ACPs), β-sheet (β-ACPs) or extended ACPs (E-ACPs) [3,36,37]. We focused our efforts using CtetPs as β-turn (U-ACPs) mimicry as innate defense regulators (IDRs) to explore novel antitumor activities. The difficult macrocyclizations for synthesizing hydrophobic CtetPs and their side-chain protected peptides was a challenging task [38,39]. In view of the wide array of bioactivities possessed by natural CtetPs, an efficient large-scale synthesis of pure products is required to evaluate the biological potential of promising bioactive compounds. Therefore, effort was directed toward the synthesis of an anionic CtetP **1** using a solution phase method. Since cationic AMP [40,41] is more common, the serine residue was then replaced with histidine providing analogous derivatives. Histidine can balance the charge and exert antibiotic activity [42,43,44]. In addition, *cyclo* (Gly-l-His-l-Pro-l-Glu) **2** must be able to cyclize from its linear precursor.

### 2.1. Cyclic Tetrapeptides Syntheses

**Scheme 1:**
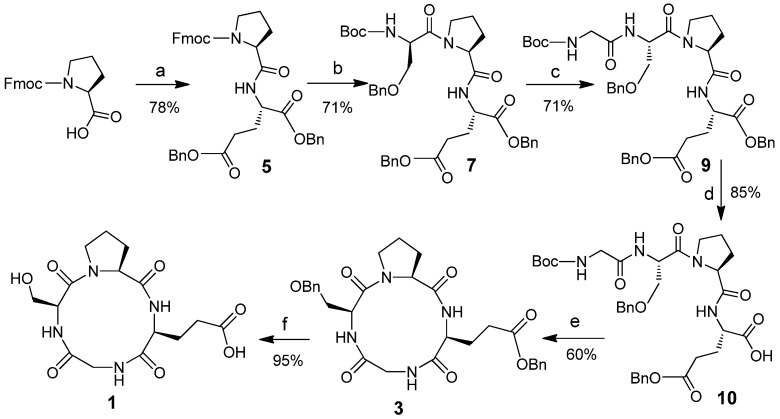
Synthesis of *cyclo*(Gly-l-Ser-l-Pro-l-Glu). **Reagents and conditions**: (**a**) *p*TSA**^.^**H_2_N-Glu-(OBn)_2_, DCC, HOBt, TEA, DCM, 0 °C–rt, overnight; (**b**) (i) 20% piperidine in DCM, (ii) Boc-Ser-OH, DCC, HOBt, TEA, DCM, 0 °C–rt, overnight; (**c**) (i) TFA, DCM, rt, (ii) Boc-Gly-OH, DCC, HOBt, TEA, DCM, 0 °C–rt; (**d**) B. *subtilis protease*, pH 7, H_2_O, acetone, 37 °C; (**e**) (i) DCC, pentafluorophenol, DCM, (ii) TFA, DCM, 0 °C, (iii) pyridine, high dilution, 90 °C, 24 h; (**f**) H_2_, Pd/C, DMF.

Dibenzyl l-glutamate toluene sulphonate [45] was used as the starting material to synthesize the CtetPs. Scheme 1 shows linear tetrapeptide Boc-GS(OBn)PE(OBn)_2_ **9** synthesized from Fmoc-l-proline. Enzymatic hydrolysis of the α-benzyl ester on glutamate was achieved regioselectively as a key step [46]. The α-carboxylic acid **10** was then activated with pentafluorophenol and cyclized with pyridine to form CtetP **3** [47]. Deprotection of the benzyl group on compound **3** completed the synthesis of CtetP **1** [19].

**Scheme 2:**
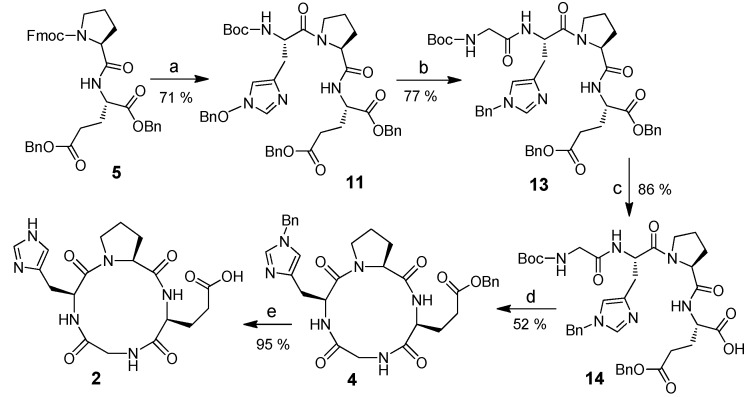
Synthesis of *cyclo*(Gly-l-His-l-Pro-l-Glu). **Reagents and conditions**: (**a**) (i) 20% piperidine in DCM, rt, (ii) Boc-His(Bn)OH, DCC, HOBt, TEA, DCM, 0 °C–rt (**b**) (i) TFA/DCM(1:1) rt, (ii) Boc-Gly-OH, DCC, HOBt, TEA, DCM, 0 °C–rt; (**c**) B. *subtilis* protease (Sigma type–VIII), pH 7, H_2_O, acetone, 37 °C; (**d**) (i) DCC, pentafluorophenol, DCM, (ii) TFA/DCM(1:1), 0 °C, (iii) pyridine, high dilution, 90 °C; (**e**) H_2_, Pd(OH)_2_/C, DMF.

The synthesis of the analogous derivatives containing histidine is shown in Scheme 2. Boc-GH(OBn)PE(OBn)_2_ (**13**) was synthesized by using a similar method starting from Fmoc-l-proline-l-glutamate dibenzyl ester (**5**). Selective hydrolysis of the α-benzyl ester on glutamate was achieved [46]. The linear precursor **14** was then activated with pentafluorophenol and cyclized with pyridine to result in CtetP **4** [47]. Compound **2** was subsequently obtained after removal of the benzyl groups.

### 2.2. Biological Activities of CtetPs

*Cyclo*(Gly-l-Ser-l-Pro-l-Glu) was active against Gram-positive *Bacillus subtilis* [31], the synthesized compounds **1**–**4** were tested for antibacterial activity against *Escherichia coli* (*E. coli*), *Staphylococcus aureus* (*S. aureus*), and *Pseudomonas aeruginosa* (*P. aer.*) assay. Table 1 shows that protected CtetP**s** indicated moderate effects against *S. aureus* (minimum inhibitory concentrations (MIC) for 24 h were mostly in the range of 60 and 120 μM). These CtetPs were not active against *E. coli* and *P. aer.*

**Table 1:** Antibacterial and antitumor activities of cyclic tetrapeptides (CtetPs). 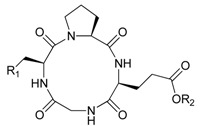

			MIC (μM) ^a^			Growth Inhibition Assay (μM) ^b^			
	*R* 1	*R* 2	*E. coli*	*P. aer.*	*S. aureus*	*A549*	*DBTRG*	*HepG2*	*LNCaP*
CtetP **1**	HO–	H	---	---	---	---	---	---	---
CtetP **2**	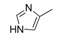	H	---	---	---	---	---	---	---
CtetP **3**	BnO–	Bn	---	---	60	108	100	---	104
CtetP **4**	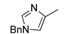	Bn	---	---	120	92	80	---	84
Tetracycline			4.5	68	1.1				
Taxol						0.0026	0.01	0.0065	0.0018

^a^ Minimal inhibitory concentrations (MICs) were defined as the lowest concentration at which no growth was detectable for 24 h. Tetracycline was used as positive control. ^b^ IC_50_ were recorded as the concentration (μM) at which cells were reduced 50% in 48 h. Taxol was used as positive control.

The His-containing CtetPs, protected or not, displayed increased cytotoxic activity against antibiotic-resistant *S. aureus* (up to 4-fold) with respect to the Ser CtetPs. However, CtetP **3**, the protected serine derivative, was the best. It is intriguing to speculate about the possible hydrophobic function on the CtetPs. The finding that benzyl protected CtetP was more effective than that without suggests that it may be particularly useful for increasing the hydrophobicity of CtetPs. Probably, the greater the hydrophobicity of the CtetPs, the greater is the antibacterial activity [48].

Surprisingly, antitumor activity against human tumor cells increased in parallel. Table 1 shows CtetP **1** and analogues which were tested for their anticancer activities towards several cancer cells using MTT assay [35]. CtetP **3** and CtetP **4** exhibited activity against three species of tumor cell, DBTRG (human brain glioblastoma multiforme), A549 (human lung adenocarcinoma epithelial), and LNCaP (human prostate carcinoma). CtetP **4** had the best results among its derivatives. These CTPs were not active against HepG2 (human hepatocellular carcinoma) cell lines. Therefore, the enhancement of peptide stability and lipophilicity improved efficacy [3,4,5,6]. Chemical modification of these compounds is promising for the development of these peptide based drugs.

### 2.3. Biological Activities of CtriPs

To obtain a low molecular weight peptide, contraction of the size of the ring is a relevant modification. Chemical synthesis of cyclic tripeptide (CtriP) was recently reported [49]. Such compounds with a 9-membered ring have never been examined for their antitumor-antibiotic activity. *Cyclo*(Gly-l-Ser-l-Pro), *cyclo*(l-Ser-l-Pro-l-Glu), and their analogues were tested for their antibiotic and anticancer activities towards several cancer cells using MTT assay [35]. None of the peptides were active in either antimicrobial or antitumor assays. The shorting of the peptide length by removal of Glu or Gly resulted in poor efficacy with concentration >200 μM. Other protected or unprotected CtPs, such as *Cyclo*(l-Ser(Bn)-l-Ser(Bn)-l-Ser(Bn)), *cyclo*(l-Ser-l-Pro-l-Ser), *Cyclo*(Gly-l-Ser(Bn)-l-Pro), *cyclo*(l-Glu-l-Glu-l-Glu), and *cyclo*(l-Glu(OBn)-l-Glu(OBn)-l-Glu(OBn)) also showed similar results. Despite this, these compounds are promising for development as enzyme inhibitors [49], due to low cytotoxicity.

### 2.4. Structure and Activity Relationship of CtetPs and CtriPs

**Figure 1:**
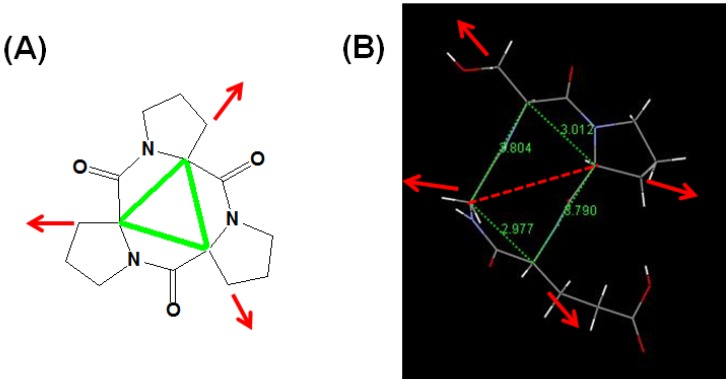
(**A**) An artifact structure of *cyclo*(l-Pro-l-Pro-l-Pro) [50] and (**B**) crystal structure of *Cyclo*(Gly-l-Ser-l-Pro-l-Glu) [19] with their Cα lines in green. The red arrow indicates the orientation of the Cα atom to the Cβ atom line.

Recently, we used the angle of the Cα atoms for a structural description of CtriPs. The angle of the Cα atoms is defined as the angle between the two lines of the Cα atom to Cα atom [48]. An example of the structure of CtriP, *Cyclo*(l-Pro-l-Pro-l-Pro) is shown in Figure 1A [50]. The angle of the Cα atoms is very close to 60° for each angle. The Cα line can be considered the mainstay of the peptide backbone. As the red arrows indicate, its side chains have to follow the core’s orientation to spread out. The line of Cα atom to Cβ atom is critical for bioactivity [44].

CtriPs and CtetPs are strained and rigid molecules; they can bind with high affinity and specificity to protein targets. The Cα atom’s angle can be used to describe the different relationship of these cyclic peptides with its membrane receptors, enzymes or functional proteins. Cα atoms’ angles that do not match the binding site are unable to bind. Through the interaction of the Cα atom’s angle, these structures are complementarily placed in the binding site of protein targets. A sharp Cα atom’s angle may be critical to fit in the binding domain. The pocket needs to be folded to nearly 300° to induce a binding with a CtriP (Figure 1A). This decreases the chance for CtriPs to obtain better inhibiting activity.

The crystal structure of *Cyclo*(Gly-l-Ser-l-Pro-l-Glu) and its Cα lines are shown in Figure 1B [19]. For CtetP, the four Cα lines are not in the same plane and not a quadrangle. A CtetP could be considered a composition of two CtriPs (two triangles), but actually all their Cα atoms’ angles are larger than 60°. We suggest that larger Cα atoms’ angles are necessary for CtetPs to become active relative to the pocket size of the target protein for antibiotic or antitumor activity. Due to the direction of the Cα atom to Cβ atom line being different to CtetP, these CtriPs are less likely to affect the proteins. This is probably the reason why CtriPs are not effective as antibiotic or antitumoral reagent.

Due to their backbone differences, CtriPs, with suitable functional groups, may facilitate the interaction with a particular protein that is not applicable to CtetPs [49], and *vice versa*. The overall clinical outcomes of the Cα atom’s angle still need to be optimized by structure modifications for a thorough investigation.

## 3. Experimental Section

### 3.1. General

All reagents were commercially available (Aldrich or Merck) and used as supplied. Solvents were dried by standard procedures. The NMR spectra were recorded on a Bruker DRX 400, (^1^H at 400.13 MHz, ^13^C at 100.03 MHz) spectrometers (Bruker Daltonics, Bremen, Germany). MALDI TOF was performed on a Bruker Autoflex MALDI-TOF mass spectrometer (Bruker Daltonnics, Breman, Germany). High-resolution electrospray ionization mass spectrometry (ESI-MS) was performed on a Shimadzu LCMS-IT-TOF mass spectrometer (Shimadzu, Kyoto, Japan). Infrared spectra were recorded on a Perkin Elmer Spectrum one FT-IR spectrometer (Perkin Elmer, Shelton, CT, USA) using KBr pellets (4000–400 cm^−1^) (Fluka, Milwaukee, WI, USA).

### 3.2. Bacterial Strains and Culture Conditions

*Staphylococcus aureus* ATCC 25923 and *Escherichia coli* ATCC 25922 were obtained from Bioresource Collection and Research Center (Hsinchu, Taiwan). *Staphylococcus aureus* was grown in Tryptic soy broth (TSB) at 37 °C. *Pseudomonas aeruginosa* and *Escherichia coli* were grown in Luria-Bertani (LB) broth at 37 °C.

### 3.3. Chemicals

Tetracycline and taxol was purchased from Sigma Aldrich (St. Louis, MO, USA). Agar and bacterial growth media such as Tryptic soy broth (TSB) and Luria-Bertani (LB) broth were purchased from Merck (Pvt. Ltd., Selangor, Malaysia).

#### 3.3.1. Fmoc-l-prolyl-l-glutamyl Dibenzyl Ester (**5**)

To a solution of triethylamine (3 g, 29.65 mmol) in dichloromethane (20 mL) in a 50 mL round bottom flask dibenzyl l-glutamyl *p*-tolunesulphonate (8.88 g, 17.79 mmol) was added at 0 °C and stirred for one hour. Then, Fmoc-l-proline (4 g, 11.86 mmol), DCC (dicyclohexylcarbodiimide) (2447 mg, 11.86 mmol) and HOBt (hydroxybenzotriazole) (1601 mg, 11.86 mmol) were added to the reaction mixture and stirred for 2 h at 0 °C and then 17 h at room temperature (rt). The resultant white mixture was filtered to remove 1,3-dicyclohexylurea (DCU). The filtrate was evaporated and the residue was dissolved in ethyl acetate. The resultant organic layer was washed with 5% aq. citric acid solution, followed by water, and then with 5% sodium bicarbonate solution followed by water. The organic layer was dried over Na_2_SO_4_. The mixture was purified by column chromatography using hexane/ethyl acetate (70:30) as eluent to obtain compound **5** as a white solid (6 g, 78%).

^1^H NMR: (CDCl_3_, 400 MHz) 1.88–1.99 (m, 4H); 2.25–2.40 (m, 4H); 3.46–3.56 (m, 2H); 4.24–4.34 (m, 3H); 4.41–4.46 (m, 2H); 4.61–4.69 (m, 1H); 5.04–5.17 (m, 4H); 7.30–7.39 (m, 14H); 7.59 (s, 2H); 7.75–7.77 (d, 2H, *J* = 7.16 Hz). ^13^C NMR: (CDCl_3_, 100 MHz) 27.2, 28.5, 301, 34.0, 47.1, 47.3, 51.8, 60.4, 66.5, 67.3, 67.7, 120.0, 125.1, 125.2, 127.1, 127.7, 128.2, 128.3, 128.5, 128.5, 128.6, 135.3, 135.8, 143.8, 144.0, 171.4, 171.7, 172.5.

#### 3.3.2. l-Prolyl-l-glutamyl Dibenzyl Ester (**6**)

To a solution of Fmoc-l-Prolyl-l-glutamyl dibenzyl ester (6 g, 9.28 mmol) in a round bottom flask a solution of 20% piperidine in dichloromethane (20 mL) was added. The reaction mixture was stirred for 2 h, concentrated, and purified by column chromatography using DCM (Dichloromethane)/MeOH (97:3) as eluent to obtain compound **6** as a pale yellow liquid (3.7 g, 94%).

^1^H NMR: (CDCl_3_, 400 MHz) 1.65–1.70 (m, 4H); 1.85–1.88 (m, 1H); 2.00–2.10 (m, 2H); 2.29–2.41 (m, 3H); 2.87–2.99 (m, 2H); 3.69–3.73 (m, 1H); 4.62–4.64 (m, 1H); 5.00–5.14 (m, 4H); 7.32 (s, 10H); 8.13–8.15 (d, 1H, *J* = 8.48 Hz). ^13^C NMR: (CDCl_3_, 100 MHz) 26.2, 27.6, 30.3, 30.9, 47.3, 51.1, 60.5, 66.5, 67.2, 128.2, 128.3, 128.4, 128.6, 128.6, 135.3, 135.8, 171.7, 172.3, 172.3, 175.4.

#### 3.3.3. Boc-l-seryl(OBn)-l-Prolyl-l-glutamate Dibenzyl Ester (**7**)

To a solution of triethylamine (1906 mg, 18.84 mmol) in dichloromethane (20 mL) in a round bottom flask compound **6** (4 g, 9.42 mmol) was added at 0 °C and stirred for 5 min. Then Boc-l-serine-OBn (2782 mg, 9.42 mmol), DCC (1944 mg, 9.42 mmol) and HOBt (1272 mg, 9.42 mmol) were added. The reaction mixture was stirred for 2 h at 0 °C and 17 h at room temperature. The resultant white mixture was filtered to remove 1,3-dicyclohexylurea. The filtrate was dried and the residue was dissolved in ethyl acetate. The organic layer was washed successively with 5% aq. citric acid solution, water, 5% aq. NaHCO_3_ solution, and water. The combined organic solvent was dried over Na_2_SO_4_ and concentrated on a rotary evaporator. The mixture was purified by column chromatography using ethyl acetate and hexane (70:30) as eluent to obtain the pure product (Yield—76%).

^1^H NMR: (CDCl_3_, 400 MHz) 1.43 (s, 9H); 1.93–2.26 (m, 8H); 3.58–3.67 (m, 4H); 4.40–4.46 (m, 3H); 4.49–4.61 (m, 2H); 5.05–5.12 (m, 4H); 7.16–7.35 (m, 15H). ^13^C NMR: (CDCl_3_, 100 MHz) 24.80, 26.96, 27.99, 28.34, 30.05, 47.68, 51.46, 51.54, 60.21, 66.35, 67.14, 71.01, 73.24, 79.99, 127.22, 127.78, 128.26, 128.36, 128.41, 128.47, 128.55, 128.57, 135.37, 135.83, 135.56, 155.17, 170.67, 170.86, 171.16, 172.29.

#### 3.3.4. l-Seryl(OBn)-l-Prolyl-l-glutamate Dibenzyl Ester (**8**)

To a round bottom flask containing compound **7** (5 mg, 7.12 mmol) 10 mL of TFA (Trifluoroacetic acid)/DCM (1:1) was added and stirred for 1 h at rt. The organic solvents were evaporated and the resultant mixture was purified by column chromatography using DCM/MeOH (97:3) as eluent to obtain compound **8** (Yield—95%).

^1^H NMR: (CDCl_3_, 400 MHz) 1.85–1.98 (m, 6H); 2.28–2.32 (m, 2H); 3.44–3.58 (m, 2H); 3.75–3.86 (m, 2H); 4.41–4.54 (m, 5H); 5.00–5.12 (m, 4H); 7.19–7.44 (m, 15H); 7.44–7.46 (d, 1H, *J* = 7.56 Hz). ^13^C NMR: (CDCl_3_, 100 MHz) 24.81, 26.59, 28.50, 30.29, 47.64, 51.86, 52.29, 60.65, 66.57, 66.97, 67.25, 73.52, 127.73, 128.15, 128.25, 128.55, 135.25, 135.66, 136.82, 132.20, 171015, 171.45, 172.98.

#### 3.3.5. Boc-glycyl-l-seryl(OBn)-l-Prolyl-l-glutamate Dibenzyl Ester (**9**)

To a solution of compound **8** (2.9 mg, 4.82 mmol) and triethylamine (975 mg, 9.64 mmol) in dichloromethane (15 mL) in a round bottom flask at 0 °C Boc-glycine-OH (845 mg, 4.82 mmol), DCC (995 mg, 4.82 mmol) and HOBt (651 mg, 4.82 mmol) were added. The reaction mixture was stirred for 2 h at 0 °C and 17 h at rt. The resultant white mixture was filtered to remove 1,3-dicyclohexylurea. The filtrate was dried and the residue was dissolved in ethyl acetate. The organic layer was washed successively with 5% aq. citric acid solution, water, 5% aq. NaHCO_3_ solution, and water. The organic layer was dried over Na_2_SO_4_ and concentrated. The resultant mixture was purified by column chromatography using DCM/MeOH (97:3) as eluent to obtain compound **9** (Yield—75%).

^1^H NMR: (CDCl_3_, 400 MHz) 1.43 (s, 9H); 1.85–1.98 (m, 6H); 2.28–2.32 (m, 2H); 3.44–3.58 (m, 2H); 3.75–3.86 (m, 2H); 4.41–4.54 (m, 5H); 5.00–5.12 (m, 4H); 7.19–7.44 (m, 15H); 7.44–7.46 (d, 1H, *J* = 7.56 Hz). ^13^C NMR: (CDCl_3_, 100 MHz) 24.74, 27.03, 28.31, 30.10, 44.01, 47.78, 50.35, 51.49, 60.28, 66.35, 67.13, 70.28, 73.28, 73.41, 80.22, 127.33, 127.81, 128.25, 128.27, 128.32, 128.42, 128.48, 128.56, 128.58, 135.37, 135.82, 137.50, 155.91, 169.12, 169.87, 170.96, 171.12, 172.24.

#### 3.3.6. Boc-glycyl-l-sery(OBn)-l-Prolyl-l-glutamic acid-α-COOH-γ-OBn (**10**)

To a solution of *B. subtilis* protease (Sigma type—VIII) (2 mg) in 0.1 M phosphate buffer (pH = 7) (3.2 mL) a solution of compound **9** (620 mg, 0.82 mmol) in 0.8 mL acetone at rt was added drop by drop. The reaction mixture was stirred at 35 °C overnight. Then the solution was basified to pH 8 to remove unchanged ester by ethyl acetate. The aqueous layer was acidified to pH 2 and centrifuged to remove enzyme. The resultant mixture was extracted with ethyl acetate and dried over Na_2_SO_4_. The organic solvent was evaporated to obtain compound **10** (Yield—85%).

^1^H NMR: (CDCl_3_, 400 MHz) 1.40 (s, 9H); 1.69–1.72 (m, 1H); 1.90–2.09 (m, 4H); 2.16–2.27 (m, 3H); 3.61–3.68 (m, 4H); 3.80–3.82 (m, 2H); 4.42–4.58 (m, 4H); 5.03–5.15 (m, 5H); 7.16–7.32 (m, 15H). ^13^C NMR: (CDCl_3_, 100 MHz) 24.78, 26.93, 28.32, 30.40, 43.72, 47.28, 48.05, 50.39, 51.66, 60.77, 66.46, 69.90, 73.35, 80.10, 127.44, 127.99, 128.27, 128.48, 128.57, 135.74, 137.25, 156.11, 169.75, 170.63, 171.39, 172.60, 172.83.

#### 3.3.7. *Cyclo*(glycyl-l-seryl(OBn)-l-Prolyl-l-glutamyl(OBn)) (**3**)

To a solution of compound **10** (200 mg, 0.29 mmol) in dichloromethane (2 mL) at 0 °C pentafluorophenol (59 mg, 0.32 mmol) and DCC (60 mg, 0.29 mmol) were added and then stirred for 1 h at 0 °C and 22 h at rt. The organic solvent was evaporated and the residue was dissolved in ethyl acetate and filtered to remove 1,3-dicyclohexylurea. The filtrate was washed with 5% aq. NaHCO_3_ solution, followed by water. The organic layer was dried over Na_2_SO_4_ to obtain pentafluorophenolic ester. The white fluffy compound was used for the next reaction without further purification.

To a solution of Boc-glycyl-l-seryl(OBn)-l-Prolyl-l-glutamic acid-γ-OBn-α-pentafluorophenolic ester (215 mg, 0.26 mmol) in dichloromethane (2 mL), TFA (2 mL) was added at 0 °C and stirred for 1 h at 0 °C. The organic solvents were evaporated; triturating with ether followed by decanting to remove free pentafluorophenol. The resultant residue was dried under vacuum*,* which was used directly in the ensuing cyclization.

To a solution of pyridine (150 mL) in a 300 mL round bottom flask at 90 °C a solution of glycyl-l-seryl(OBn)-l-Prolyl-l-glutamic acid-γ-OBn-α-pentafluorophenolic ester (200 mg, 0.272 mmol) in dried 1-4 dioxane (10 mL) was added drop wise with efficient stirring. Addition was completed after 6 h. After stirring for 24 h, the solvent was distilled off. The resultant residue was treated several times with ether and finally methanol was added to precipitate the product. The precipitate was washed with 1 N HCl, water and finally with methanol and dried in vacuum to obtain compound **3** as a white solid (overall Yield—60%).

^1^H NMR: (CD_3_OD & CDCl_3_, 400 MHz) 1.62–1.76 (m, 3H); 1.98–2.03 (m, 3H); 2.15–2.26 (m, 2H); 3.25–3.9 (m, 2H); 3.49–3.69 (m, 2H); 3.75–3.90 (m, 2H); 4.18–4.20 (d, 1H, *J* = 8 Hz); 4.27 (d, 2H, *J* = 14.4 Hz); 4.37 (d, 1H, *J* = 13,2 Hz); 4.52 (s, 1H); 4.70 (s, 1H); 4.89–4.96 (m, 2H); 7.10–7.17 (m, 10H). MALDI-TOF: *m*/*z* 551.38 [M + H]^+^.

#### 3.3.8. *Cyclo*(glycyl-l-seryl-l-Prolyl-l-glutamyl) (**1**)

To a solution of compound **3** (100 mg) in DMF (5 mL), 10% palladium on carbon (10 mg) was added under nitrogen. The vessel was purged three times with nitrogen and three times with hydrogen and the mixture was then stirred for 6 h under hydrogen at atmospheric pressure. The catalyst was removed by filtration and the filtrate was evaporated to obtain **1** as a white solid (Yield—95%).

^1^H NMR: (D_2_O, 400 MHz) 1.60–1.89 (m, 3H); 1.93–2.17 (m, 3H); 2.20–2.38 (m, 2H); 3.36–3.54 (m, 2H); 3.57–3.65 (m, 1H); 3.77–3.82 (m, 1H); 3.86–3.97 (m, 2H); 4.08–4.10 (d, 1H, *J* = 8 Hz ); 4.35–4.37 (d, 1H, *J* = 8 Hz ); 4.80–4.82 (m, 1H).

IR (cm^−1^): 3459, 3333, 3294, 3236, 3066, 2990, 2961, 1718, 1663, 1646, 1615, 1545, 1444, 1380, 1335, 1285, 1242, 1222, 1161, 1138, 1108, 1056, 1042, 978, 925, 875, 776, 758, 679, 605, 583, 482. MALDI-TOF: *m*/*z* 371.35 [M + H]^+^, ESI-MS: [M − H]^−^ calculated *m*/*z* 369.1488, obtained *m*/*z* 369.1426.

#### 3.3.9. Boc-l-histidinyl(Bn)-l-Prolyl-l-glutamyl Dibenzyl Ester (**11**)

To a solution of triethylamine (1906 mg, 18.84 mmol) in dichloromethane (20 mL) in a round bottom flask, Boc-l-histidine(Bn) (3255 mg, 9.42 mmol), DCC (1944 mg, 9.42 mmol) and HOBt (1272 mg, 9.42 mmol) were added at 0 °C. Then l-proline-l-glutamyl dibenzyl ester 6 (4 g, 9.42 mmol) was added. The reaction mixture was stirred for 2 h at 0 °C and 17 h at rt. The resultant white mixture was filtered to remove 1,3-dicyclohexylurea. The filtrate was dried and the residue was dissolved in ethyl acetate. The organic layer was washed successively with 5% aq. citric acid solution, water, 5% aq. NaHCO_3_ solution, and water. The combined organic layer was dried over Na_2_SO_4_ and purified by column chromatography using DCM/MeOH (97:3) as eluent to obtain compound **11** as a white solid (5.4 g, 76%).

^1^H NMR: (CDCl_3_, 400 MHz) 1.41 (s, 9H); 1.68–1.74 (m, 2H); 1.99–2.10 (m, 2H); 2.20–2.52 (m, 4H); 2.86–2.88 (d, 2H, *J* = 8 Hz); 3.35–3.41 (m, 1H); 4.43–4.51 (m, 2H); 4.58–4.60 (m, 1H); 4.93 (s, 2H); 4.95–5.07 (m, 2H); 5.13 (q, 2H); 5.51–5.53 (d, 2H, *J* = 7.96 Hz); 6.72 (s, 1H); 7.09–7.11 (m, 2H); 7.28–7.35 (m, 15H); 9.34–9.36 (d, 2H, *J* = 8 Hz). ^13^C NMR: (CDCl_3_, 100 MHz) 24.7, 26.0, 28.4, 29.0, 30.5, 32.2, 47.2, 50.9, 51.8, 52.7, 60.8, 66.2, 67.0, 79.6, 117.6, 127.3, 128.1, 128.2, 128.3, 128.4, 128.5, 128.5, 129.1, 136.0, 136.9, 137.8, 155.1, 171.3, 171.4, 171.8, 172.9.

#### 3.3.10. l-Histidinyl(Bn)-l-Prolyl-l-glutamyl Dibenzyl Ester (**12**)

To a round bottom flask containing Boc-l-histidinyl(Bn)-l-Prolyl-l-glutamyl dibenzyl ester **11** (5.4 g, 7.19 mmol), 10 mL of TFA/DCM (1:1) was added and stirred for 1 h at rt. The organic solvents were evaporated and the resultant mixture was purified by column chromatography using DCM/MeOH (97:3) as eluent to obtain compound **12** as a pale yellow liquid (4.3g, 93%).

^1^H NMR: (CDCl_3_, 400 MHz) 1.84–1.86 (m, 3H); 2.05–2.25 (m, 3H); 2.42 (t, 2H, *J* = 7.44 Hz); 3.15–3.56 (m, 3H); 3.57–3.60 (m, 1H); 4.50–4.51 (m, 3H); 5.00–5.12 (m, 4H); 5.00 (s, 2H); 5.02–5.12 (m, 4H); 7.21–7.34 (m, 15H); 8.04 (s, 1H); 8.56–8.58 (d, 1H, *J* = 6.12 Hz). ^13^C NMR: (CDCl_3_, 100 MHz) 24.9, 26.3, 29.2, 30.3, 31.0, 47.6, 51.2, 52.1, 52.5, 60.7, 66.4, 67.0, 115.2, 118.1, 121.6, 128.1, 128.1, 128.4, 128.5, 129.3, 133.7, 135.4, 135.8, 162.0, 167.2, 171.4, 172.9.

#### 3.3.11. Boc-glycyl-l-histidinyl(Bn)-l-Prolyl-l-glutamyl Dibenzyl Ester (**13**)

To a solution of l-histidinyl(Bn)-l-Prolyl-l-glutamyl dibenzyl ester **12** (4.3 g, 6.6 mmol) and triethylamine (1336 mg, 13.3 mmol) in dichloromethane (20 mL) in a round bottom flask, Boc-glycine (1156 mg, 6.6 mmol), DCC (1362 mg, 6.6 mmol) and HOBt (891 mg, 6.6 mmol) were added at 0 °C. The reaction mixture was stirred for 2 h at 0 °C and 17 h at rt. The resultant white mixture was filtered to remove 1,3-dicyclohexylurea. The filtrate was evaporated and the residue was dissolved in ethyl acetate. The organic layer was washed successively with 5% aq. citric acid solution, water, 5% aq. NaHCO_3_ solution and water. The organic layer was dried over Na_2_SO_4_ and concentrated. The resultant mixture was purified by column chromatography using DCM/MeOH (97:3) as eluent to obtain compound **13** as a white solid (4.4 g, 83%).

^1^H NMR: (CDCl_3_, 400 MHz) 1.42 (s, 9H); 1.66–1.76 (m, 2H); 1.97–2.08 (m, 2H); 2.16–3.49 (m, 4H); 2.84–2.98 (m, 3H); 3.44 (q, 1H); 3.77–3.78 (d, 2H, *J* = 4.96 Hz); 4.48 (q, 1H); 4.56 (dd, 1H, *J* = 2.76 Hz, *J* = 8.44 Hz); 4.73–4.78 (m, 1H); 4.92 (s, 2H); 4.95–5.12 (m, 4H); 5.23 (s, 1H); 6.73 (s, 1H); 7.09–7.11 (m, 2H); 7.23–7.34 (m, 15H); 9.24–9.26 (d, 1H, *J* = 7.48 Hz). ^13^C NMR: (CDCl_3_, 100 MHz) 24.6, 26.1, 28.3, 29.1, 30.5, 31.5, 44.0, 47.4, 50.9, 51.5, 51.8, 60.8, 66.2, 66.9, 80.1, 117.8, 127.4, 128.1, 128.2, 128.2, 128.4, 128.5, 128.5, 129.0, 135.7, 135.8, 136.0, 136.9, 137.4, 155.9, 168.7, 170.8, 171.4, 171.8, 172.8.

#### 3.3.12. Boc-glycyl-l-histidinyl(Bn)-l-Prolyl-l-glutamic acid-γ(OBn) (**14**)

To a solution of B. *subtilis* protease (Sigma type—VIII) (50 mg) in 32 mL of 0.1 M, pH 7 phosphate buffer, a solution of Boc-glycyl-l-histidinyl(Bn)-l-Prolyl-l-glutamyl dibenzyl ester **13** (4.4 g, 5.44 mmol) in acetone (8 mL) was added drop by drop. The reaction mixture was stirred at 35 °C overnight. Then the solution was basified to pH 8 to remove unchanged ester with ethyl acetate. The aqueous layer was acidified to pH 2 and centrifuged to remove enzyme. The resultant mixture was extracted with ethyl acetate and dried over Na_2_SO_4_. The organic portion was evaporated and purified by column chromatography using DCM/MeOH (97:3) as eluent to obtain compound **14** as a white solid (3.36 g, 86%).

^1^H NMR: (CD_3_OD, 400 MHz) 1.42 (s, 9H); 1.90–2.01 (m, 4H); 2.20–2.29 (m, 2H); 2.47–2.55 (m, 2H); 2.98 (dd, 1H, *J* = 5.6 Hz, *J* = 15.08 Hz); 3.17 (dd, 1H, *J* = 6.8 Hz, *J* = 15.04 Hz); 3.45–3.52 (m, 1H); 3.62 (s, 2H); 4.44 (q, 1H); 4.50 (q, 1H); 4.93 (s, 1H); 5.09 (q, 2H); 5.34 (s, 2H); 7.23–7.34 (m, 15H); 7.40 (s, 1H); 8.88 (s, 1H). ^13^C NMR: (CD_3_OD, 100 MHz) 24.6, 26.4, 26.6, 27.3, 29.3, 29.9, 43.0, 50.0, 51.5, 52.5, 60.2, 63.8, 66.0, 79.4, 110.0, 117.2, 120.8, 127.0, 127.8, 127.8, 128.0, 128.2, 128.3, 129.0, 129.0, 134.0, 134.5, 136.1, 141.3, 168.5, 170.7, 172.8, 173.1, 173.4.

#### 3.3.13. *Cyclo*(glycyl-l-histidinyl(Bn)-l-Prolyl-l-glutamyl(OBn) (**4**)

A similar procedure as for the synthesis of compound **3** was applied to obtain compound **4** as a white solid with an overall yield of 52%.

^1^H NMR: (CD_3_OD & CDCl_3_, 400 MHz) 143–1.63 (m, 3H); 1.92 (s, 2H); 2.02–2.30 (m, 3H); 2.75–2.89 (m, 1H); 2.96–2.99 (m, 1H); 3.35–3.48 (m, 3H); 3.71 (s, 2H); 4.68 (s, 1H); 4.73–4.75 (m, 1H); 4.86–4.93 (m, 2H); 5.07–5.17 (m, 2H); 6.92 (s, 1H); 7.14–7.26 (m, 10H); 8.49 (s, 1H). ^13^C NMR: (CD_3_OD & CDCl_3_, 100 MHz) 25.4, 25.9, 30.3, 33.6, 35.8, 49.3, 51.5, 51.6, 56.7, 56.9, 65.6, 70.3, 122.5, 124.2, 132.0, 132.1, 132.1, 132.2, 132.3, 132.4, 133.2, 133.3, 135.6, 137.0, 139.7, 173.4, 174.3, 176.1, 176.7, 178.3. MALDI-TOF: [M + H]^+^ calculated *m*/*z* 601.277, obtained *m*/*z* 601.383.

#### 3.3.14. Cyclo(glycyl-l-histidinyl-l-Prolyl-l-glutamyl) (**2**)

*Cyclo*(glycyl-l-histidinyl(Bn)-l-Prolyl-l-glutamyl(OBn) **4** (100 mg, 0.17 mmol) was dissolved in 5 mL of DMF and 10% palladium hydroxide on carbon was added under nitrogen. The vessel was purged three times with nitrogen and then the bottle was shaken at 50 lbs per sq inch hydrogen pressure in a Parr shaker for 8 h. The catalyst was removed by filtration and the filtrate was evaporated to obtain compound **2** as a white powder (77 mg, 95%) [α]^21^_D_ = −117.75 (C = 0.1, H_2_O).

^1^H NMR: (D_2_O, 400 MHz) 1.56–2.02 (m, 5H); 2.16 (s, 3H); 2.83–2.93 (m, 1H); 3.10–3.20 (m, 1H); 3.39–3.58 (m, 2H); 3.63–3.75 (m, 2H); 3.80–3.95 (m, 1H); 4.08 (dd, 1H, *J* = 8.56 Hz, *J* = 40.16 Hz); 4.88–4.98 (m, 1H); 7.14 (s, 1H); 8.44 (s,1H). ^13^C NMR: (D_2_O, 100 MHz) 21.3, 24.2, 26.0, 30.8, 31.5, 46.0, 48.0, 50.3, 51.8, 59.5, 117.4, 129.0, 133.0, 168.8, 170.6, 171.1, 173.8, 175.6. IR (cm^−1^): 3272.12, 1668.93, 1541.19, 1443.98, 1346.04, 1205.54, 1135.58, 981.93, 838.79, 799.63, 749.84, 722.56, 670.75, 629.16, 604.80, 477.38. MALDI-TOF: [M + H]^+^ calculated *m*/*z* 421.183, obtained *m*/*z* 421.296. HRMS (ESI): [M + H]^+^ calculated: *m*/*z* 421.1830, obtained: *m*/*z* 421.1841.

### 3.4. Minimal Inhibitory Concentrations (MICs) Assay

The antibacterial activity of the drug against *Staphylococcus aureus*, *Pseudomonas aeruginosa*, and *Escherichia coli* were determined by the usual twofold dilution technique with Mueller-Hinton agar (Merck Millipore, Darmstadt, Germany) and an inoculum size of 104 cells per mL according to the guidelines of standard procedures. Strains were grown in TSB or LB broth to a turbidity of 0.5 McFarland standards and, using sterile cotton swabs, the bacterial inoculum were added into tubes by lawn culture. Drug serial dilutions were carefully loaded onto the tubes. Tetracycline antibiotic was included as an antibiotic control. All tubes were incubated at 37 °C for 24 h until visible growth was observed and the optical densities were measured and compared with the control antibiotic. The experiment was performed in duplicate.

### 3.5. Cell Lines and Culture

LNCaP, A549, HepG2, and DBTRG were obtained from ATCC (American Type Culture Collection, Manassas, VA, USA). LNCaP, and A549, were maintained with RPMI 1640 medium containing 10% fetal bovine serum, 100 U/mL penicillin, 100 U/mL streptomycin, 1% sodium pyruvate, and 1% HEPES. DBTRG was maintained with DMEM medium containing 10% fetal bovine serum, 100 U/mL penicillin, 100 U/mL streptomycin, 1% sodium pyruvate, and 1% sodium bicarbonate. All of the cells were at 37 °C in a humidified atmosphere with 5% CO_2_. All cultures were free of Mycoplasma.

### 3.6. MTT Assay for Cancer Cell Lines (Growth Inhibition Assay)

The viability of the cells after treatment with various chemicals was evaluated using an MTT assay preformed in triplicate. The cancer cells (5 × 10^3^) were incubated in 96-well plates containing 200 μL of serum-containing medium. Cells were permitted to adhere for 12–18 h. Solutions were always prepared fresh by dissolving 0.2% DMSO or drugs in culture medium and added to cancer cells. After 24 h and 48 h of exposure, the drug-containing medium was removed. The cells in each well were then incubated in culture medium with 500 μg/mL MTT for 1 h. Absorbance at 595 nm of the maximum, was detected by an ELISA Reader (Bio-Rad, Hercules, CA, USA).

## 4. Conclusions

The antibacterial and antitumor activities of analogue peptides based on cyclic tetramer and trimer were described. The modified peptides showed activity against *S. aureus* in the range of 60–120 μM (24 h). Especially, benzyl group protected *cyclo*(Gly-l-Ser-l-Pro-l-Glu) exhibited the strongest activities against antibiotic-resistant *S. aureus* while no cytotoxicity was observed toward gram negative *E coli* and *P. aer*. This study also suggests that the hydrophobic protecting group is a useful technique for improving antibiotic activity without increasing resistance or reducing susceptibility to proteolysis. In addition, benzyl protected CtetPs showed an improved activity against tumor cells compared to those without. The poor efficacy of CtriPs and their low cytotoxicity was disclosed. This is probably due to lack of β-turn (U-ACPs) mimicry, orientation of their side-chain group and/or their chemical stability. The present findings also suggest that the potential of hydrophobic group protected cyclic tetrapeptides should not be overlooked when contemplating the design of both antitumor and antimicrobial properties. Further research may promote the great potential of linear peptide linked β-turn (U-ACPs) mimicry [28] for the development of peptide-based antitumor-antibiotic drugs.

## Supplementary Information

Synthetic methods and spectral data ( ^1^H, ^13^C NMR, IR, MS) for cyclic tetrapeptides and their precursors.
