# Supplementary material for: Antitumor and Antimicrobial Activity of Some Cyclic Tetrapeptides and Tripeptides Derived from Marine Bacteria
Source: Mar Drugs. 2015 May 15;13(5):3029–45. doi: 10.3390/md13053029 (PMC4446616; doi:10.3390/md13053029)
Supplement: Supplementary file 1 [file marinedrugs-13-03029-s001.pdf]

Supplementary Information

Contents	
Experimental procedure	S1–S9
Spectra (Figures S15–S44)	S10–S39

1. Experimental Procedure

*1.1. Fmoc-L-prolyl-L-glutamyl Dibenzyl Ester (**5**)*

To a solution of triethylamine (3 g, 29.65 mmol) in dichloromethane (20 mL) in a 50 mL round bottom flask was added dibenzyl L-glutamyl *p*-tolunesulphonate[25] (8.88 g, 17.79 mmol) at 0 °C and stirred for one hour. Then, Fmoc-L-proline (4 g, 11.86 mmol), DCC (2447 mg, 11.86 mmol) and HOBt (1601 mg, 11.86 mmol) were added to the reaction mixture and stirred for 2 h at 0 °C and then 17 h at room temperature. The resultant white mixture was filtered to remove 1, 3-dicyclohexylurea (DCU). The filtrate was evaporated and the residue was dissolved in ethyl acetate. The resultant organic layer was washed with 5% aq. citric acid solution followed by water and then with 5% sodium bicarbonate solution followed by water. The organic layer was dried over Na_2SO_4. The mixture was purified by column chromatography using hexane/ethyl acetate (70:30) as eluent to obtain compound **5** (Figure S1) as a white solid (6 g, 78%).

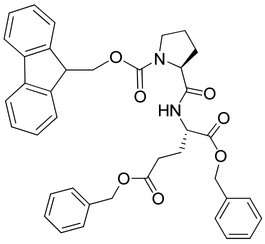

(**5**)

Figure S1. Fmoc-L-prolyl-L-glutamyl Dibenzyl Ester.

^{1}H NMR: ($CDCl_3$, 400 MHz) 1.88–1.99 (m, 4H); 2.25–2.40 (m, 4H); 3.46–3.56 (m, 2H); 4.24–4.34 (m, 3H); 4.41–4.46 (m, 2H); 4.61–4.69 (m, 1H); 5.04–5.17 (m, 4H); 7.30–7.39 (m, 14H); 7.59 (s, 2H); 7.75–7.77 (d, 2H, J = 7.16 Hz). ^{13}C NMR: ($CDCl_3$, 100 MHz) 27.2, 28.5, 301, 34.0, 47.1, 47.3, 51.8, 60.4, 66.5, 67.3, 67.7, 120.0, 125.1, 125.2, 127.1, 127.7, 128.2, 128.3, 128.5, 128.5, 128.6, 135.3, 135.8, 143.8, 144.0, 171.4, 171.7, 172.5.

*1.2. L-Prolyl-L-glutamyl Dibenzyl Ester (**6**)*

To a solution of Fmoc-L-prolyl-L-glutamyl dibenzyl ester (6 g, 9.28 mmol) in a round bottom flask was added a solution of 20% piperidine in dichloromethane (20 mL). The reaction mixture was stirred for 2 h, concentrated and purified by column chromatography using DCM/MeOH (96:4) as eluent to obtain compound **6** (Figure S2) as a pale yellow liquid (3.7 g, 94%).

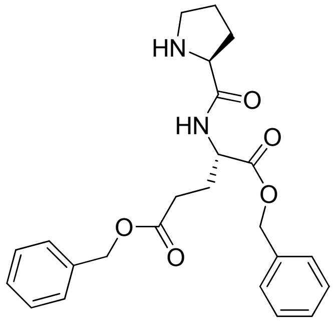

(**6**)

Figure S2. L-Prolyl-L-glutamyl Dibenzyl Ester.

^{1}H NMR: ($CDCl_3$, 400 MHz) 1.65–1.70 (m, 4H); 1.85–1.88 (m, 1H); 2.00–2.10 (m, 2H); 2.29–2.41 (m, 3H); 2.87–2.99 (m, 2H); 3.69–3.73 (m, 1H); 4.62–4.64 (m, 1H); 5.00–5.14 (m, 4H); 7.32 (s, 10H); 8.13–8.15 (d, 1H, *J* = 8.48 Hz). ^{13}C NMR: ($CDCl_3$, 100 MHz) 26.2, 27.6, 30.3, 30.9, 47.3, 51.1, 60.5, 66.5, 67.2, 128.2, 128.3, 128.4, 128.6, 128.6, 135.3, 135.8, 171.7, 172.3, 172.3, 175.4.

1.3. Boc-L-seryl(OBn)-L-prolyl-L-glutamate Dibenzyl Ester (7)

To a solution of triethylamine (1906 mg, 18.84 mmol) in dichloromethane (20 mL) in a round bottom flask was added compound **6** (4 g, 9.42 mmol) at 0 °C and stirred for 5 minutes. Then Boc-L-serine-OBn (2782 mg, 9.42 mmol), DCC (1944 mg, 9.42 mmol) and HOBt (1272 mg, 9.42 mmol) were added. The reaction mixture was stirred for 2 h at 0 °C and 17 h at room temperature. The resultant white mixture was filtered to remove 1,3-dicyclohexylurea. The filtrate was dried and residue was dissolved in ethyl acetate. The organic layer was washed successively with 5% aq. citric acid solution, water, 5% aq. $NaHCO_3$ solution and water. The combined organic solvent was dried over Na_2SO_4 and concentrated on rotary evaporator. The mixture was purified by column chromatography using ethyl acetate and hexane (65:35) as eluent to obtain compound **7** (Figure S3) as white solid (5 g, 76%).

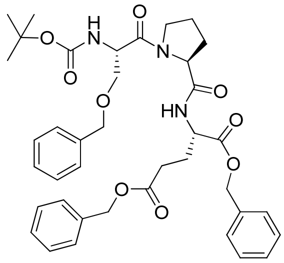

(**7**)

Figure S3. Boc-L-seryl(OBn)-L-prolyl-L-glutamate dibenzyl ester.

^{1}H NMR: ($CDCl_3$, 400 MHz) 1.43 (s, 9H); 1.93–2.26 (m, 8H); 3.58–3.67 (m, 4H); 4.40–4.46 (m, 3H); 4.49–4.61 (m, 2H); 5.05–5.12 (m, 4H); 7.16–7.35 (m, 15H). ^{13}C NMR: ($CDCl_3$, 100 MHz) 24.80, 26.96, 27.99, 28.34, 30.05, 47.68, 51.46, 51.54, 60.21, 66.35, 67.14, 71.01, 73.24, 79.99, 127.22, 127.78, 128.26, 128.36, 128.41, 128.47, 128.55, 128.57, 135.37, 135.83, 135.56, 155.17, 170.67, 170.86, 171.16, 172.29.

*1.4. L-Seryl(OBn)-L-prolyl-L-glutamate dibenzyl ester (**8**)*

To a round bottom flask containing compound **7** (5 gm, 7.12 mmol) was added 10 mL of TFA/DCM (1:1) and stirred for 1 h at RT. The organic solvents were evaporated and the resultant mixture was purified by column chromatography using DCM/MeOH (96:4) as eluent to obtain compound **8** (Figure S4) as pale yellow liquid (4 g, 95%).

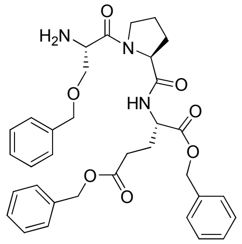

(**8**)

Figure S4. L-Seryl(OBn)-L-prolyl-L-glutamate dibenzyl ester.

^{1}H NMR: ($CDCl_3$, 400 MHz) 1.85–1.98 (m, 6H); 2.28–2.32 (m, 2H); 3.44–3.58 (m, 2H); 3.75–3.86 (m, 2H); 4.41–4.54 (m, 5H); 5.00–5.12 (m, 4H); 7.19–7.44 (m, 15H); 7.44–7.46 (d, 1H, J = 7.56 Hz). [13] C NMR : ($CDCl_3$, 100 MHz) 24.81, 26.59, 28.50, 30.29, 47.64, 51.86, 52.29, 60.65, 66.57, 66.97, 67.25, 73.52, 127.73, 128.15, 128.25, 128.55, 135.25, 135.66, 136.82, 132.20, 171015, 171.45, 172.98.

*1.5. Boc-glycyl-L-seryl(OBn)-L-prolyl-L-glutamate Dibenzyl Ester (**9**)*

To a solution of compound **8** (2.9 gm, 4.82 mmol) and triethylamine (975 mg, 9.64 mmol) in dichloromethane (15 mL) in a round bottom flask at 0 °C was added Boc-glycine-OH (845 mg, 4.82 mmol), DCC (995 mg, 4.82 mmol) and HOBt (651 mg, 4.82 mmol). The reaction mixture was stirred for 2 h at 0 °C and 17 h at RT (room temperature). The resultant white mixture was filtered to remove 1,3-dicyclohexylurea. The filtrate was dried and the residue was dissolved in ethyl acetate. The organic layer was washed successively with 5% aq. citric acid solution, water, 5% aq. $NaHCO_3$ solution and water. The organic layer was dried over Na_2SO_4 and concentrated. The resultant mixture was purified by column chromatography using DCM/MeOH (97:3) as eluent to obtain compound **9** (Figure S5) as a white solid (2.7 g, 75%).

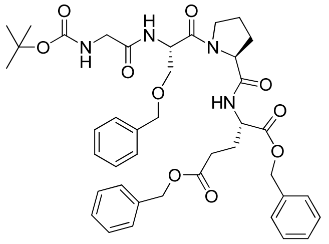

(**9**)

Figure S5. Boc-glycyl-L-seryl(OBn)-L-prolyl-L-glutamate dibenzyl ester.

^{1}H NMR: ($CDCl_3$, 400 MHz) 1.43 (s, 9H); 1.85–1.98 (m, 6H); 2.28–2.32 (m, 2H); 3.44–3.58 (m, 2H); 3.75–3.86 (m, 2H); 4.41–4.54 (m, 5H); 5.00–5.12 (m, 4H); 7.19–7.44 (m, 15H); 7.44–7.46 (d, 1H,

J = 7.56 Hz). [13] C NMR: ($CDCl_3$, 100 MHz) 24.74, 27.03, 28.31, 30.10, 44.01, 47.78, 50.35, 51.49, 60.28, 66.35, 67.13, 70.28, 73.28, 73.41, 80.22, 127.33, 127.81, 128.25, 128.27, 128.32, 128.42, 128.48, 128.56, 128.58, 135.37, 135.82, 137.50, 155.91, 169.12, 169.87,170.96, 171.12, 172.24.

*1.6. Boc-glycyl-L-sery(OBn)-L-prolyl-L-glutamic Acid-α-COOH -γ-OBn (**10**)*

To a solution of B. *subtilis* protease (Sigma type–VIII) (2 mg) in 0.1 M phosphate buffer (pH = 7) (3.2 mL) was added drop by drop a solution of compound **9** (620 mg, 0.82 mmol) in 0.8 mL acetone at RT. The reaction mixture was stirred at 35 °C over night. Then the solution was basified to 8 to remove unchanged ester by ethyl acetate. The aqueous layer was acidified to pH 2 and centrifuged to remove enzyme. The resultant mixture was extracted with ethyl acetate and dried over Na_2SO_4. The organic solvent was evaporated to obtain compound **10** (Figure S6) as a white solid (466 mg, 85%).

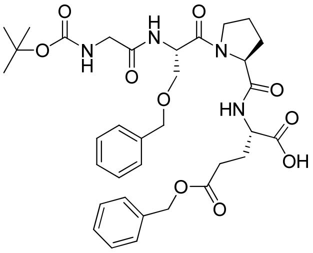

(**10**)

Figure S6. Boc-glycyl-L-sery(OBn)-L-prolyl-L-glutamic acid-α-COOH -γ-Obn.

^{1}H NMR: ($CDCl_3$, 400 MHz) 1.40 (s, 9H); 1.69–1.72 (m, 1H); 1.90–2.09 (m, 4H); 2.16–2.27 (m, 3H); 3.61–3.68 (m, 4H); 3.80–3.82 (m, 2H); 4.42–4.58 (m, 4H); 5.03–5.15 (m, 5H); 7.16–7.32 (m, 15H). ^{13}C NMR: ($CDCl_3$, 100 MHz) 24.78, 26.93, 28.32, 30.40, 43.72, 47.28, 48.05, 50.39, 51.66, 60.77, 66.46, 69.90, 73.35, 80.10, 127.44, 127.99, 128.27, 128.48, 128.57, 135.74, 137.25, 156.11, 169.75, 170.63, 171.39, 172.60, 172.83.

*1.7. Cyclo(glycyl-L-seryl(OBn)-L-prolyl-L-glutamyl(OBn)) (**3**)*

To a solution of compound **10** (200 mg, 0.29 mmol) in dichloromethane (2 mL) at 0 °C was added pentafluorophenol (59 mg, 0.32 mmol) and DCC (60 mg, 0.29 mmol) and stirred for 1 h at 0 °C and 22 h at RT. The organic solvent was evaporated and the residue was dissolved in ethyl acetate and filtered to remove 1,3-dicyclohexylurea. The filtrate was washed with 5% aq. $NaHCO_3$ solution, followed by water. The organic layer was dried over Na_2SO_4 to obtain pentafluoro-phenol ester. The white compound was used for the next reaction without further purification.

To a solution of Boc-glycyl-L-seryl(OBn)-L-prolyl-L-glutamic acid-γ-OBn-α-pentafluoro-phenol ester (215 mg, 0.26 mmol) in dichloromethane (2 mL) was added TFA (2 mL) at 0 °C and stirred for 1 h at 0 °C. The organic solvents were evaporated; triturating with ether followed by decanting removed free pentafluorophenol. The resultant residue was dried in vacuum*,* which was used directly in the ensuing cyclization.

To a solution of pyridine (150 mL) in a 300 mL round bottom flask at 90 °C was added drop wise a solution of glycyl-L-seryl(OBn)-L-prolyl-L-glutamic acid-γ-OBn-α-pentafluoro-phenol ester (200 mg,

0.272 mmol) in dried 1-4 dioxane (10 mL) with efficient stirring. Addition was completed after 6 h. After stirring over 24 h, the solvent was distilled off. The resultant residue was treated several times with ether and finally methanol was added to precipitate out the product. The precipitate was washed with 1 N HCl, water and finally with methanol and dried in vacuum to obtain compound **3** (Figure S7) as a white solid (120 mg, 60%).

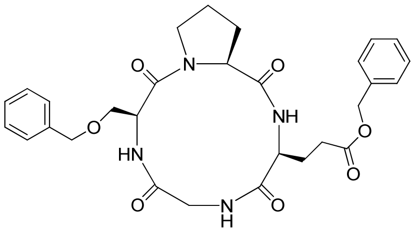

(**3**)

Figure S7. Cyclo(glycyl-L-seryl(OBn)-L-prolyl-L-glutamyl(OBn)).

^{1}H NMR: (CD_3OD & $CDCl_3$, 400 MHz) 1.62–1.76 (m, 3H); 1.98–2.03 (m, 3H); 2.15–2.26 (m, 2H); 3.25–3.9 (m, 2H); 3.49–3.69 (m, 2H); 3.75–3.90 (m, 2H); 4.18–4.20 (d, 1H, J = 8 Hz); 4.26–4.38 (dd, 2H, J = 14.6 Hz J = 37.76 Hz); 4.52 (s, 1H); 4.70 (s, 1H); 4.89–4.96 (m, 2H); 7.10–7.17 (m, 10H). MALDI-TOF: *m/z* 551.38 $[M + H]^+$

*1.8. Cyclo(glycyl-L-seryl-L-prolyl-L-glutamyl) (**1**)*

To a solution of compound **3** (100 mg) in DMF (5 mL) was added 10% palladium on carbon (10 mg) under nitrogen. The vessel was purged three times with nitrogen and three times with hydrogen and the mixture was then stirred for 6 h under hydrogen at atmospheric pressure. The catalyst was removed by filtration and the filtrate was evaporated to obtain **1** (Figure S8) as a white solid (64 mg, 95%).

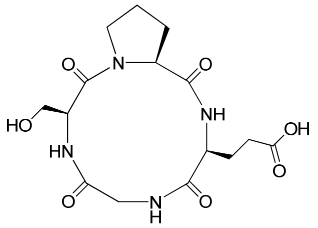

(**1**)

Figure S8. Cyclo(glycyl-L-seryl-L-prolyl-L-glutamyl).

^{1}H NMR: (D_2O, 400 MHz) 1.60–1.89 (m, 3H); 1.93–2.17 (m, 3H); 2.20–2.38 (m, 2H); 3.36–3.54 (m, 2H); 3.57–3.65 (m, 1H); 3.77–3.82 (m, 1H); 3.86–3.97 (m, 2H); 4.08–4.10 (d, 1H, J = 8 Hz); 4.35–4.37 (d, 1H, J = 8 Hz); 4.80–4.82 (m, 1H).

IR (cm^{-1}): 3459, 3333, 3294, 3236, 3066, 2990, 2961, 1718, 1663, 1646, 1615, 1545, 1444, 1380, 1335, 1285, 1242, 1222, 1161, 1138, 1108, 1056, 1042, 978, 925, 875, 776, 758, 679, 605, 583, 482. MALDI-TOF: *m/z* 371.35 $[M + H]^+$, ESI-MS: $[M - H]^-$ calculated *m/z* 369.1488, obtained *m/z* 369.1426.

*1.9. Boc-L-histidinyl(OBn)-L-prolyl-L-glutamyl Dibenzyl Ester (**11**)*

To a solution of triethylamine (1906 mg, 18.84 mmol) in dichloromethane (20 mL) in a round bottom flask was added Boc-L-histidine(OBn) (3255 mg, 9.42 mmol), DCC (1944 mg, 9.42 mmol) and HOBt (1272 mg, 9.42 mmol) at 0 °C. Then L-proline-L-glutamyl dibenzyl ester **6** (4 g, 9.42 mmol) was added. The reaction mixture was stirred for 2 h at 0 °C and 17 h at RT. The resultant white mixture was filtered to remove 1, 3-dicyclohexylurea. The filtrate was dried and the residue was dissolved in ethyl acetate. The organic layer was washed successively with 5% aq. citric acid solution, water, 5% aq. $NaHCO_3$ solution and water. The combined organic layer was dried over Na_2SO_4 and purified by column chromatography using DCM/MeOH (96.5:3.5) as eluent to obtain compound **11** (Figure S9) as a white solid (5.4 g, 76%).

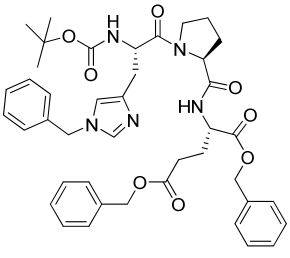

(**11**)

Figure S9. Boc-L-histidinyl(OBn)-L-prolyl-L-glutamyl dibenzyl ester.

^{1}H NMR: ($CDCl_3$, 400 MHz) 1.41 (s, 9H); 1.68–1.74 (m, 2H); 1.99–2.10 (m, 2H); 2.20–2.52 (m, 4H); 2.86–2.88 (d, 2H, J = 8 Hz); 3.35–3.41 (m, 1H); 4.43–4.51 (m, 2H); 4.58–4.60 (m, 1H); 4.93 (s, 2H); 4.95–5.07 (m, 2H); 5.13 (q, 2H); 5.51–5.53 (d, 2H, J = 7.96 Hz); 6.72 (s, 1H); 7.09–7.11 (m, 2H); 7.28–7.35 (m, 15H); 9.34–9.36 (d, 2H, J = 8 Hz). ^{13}C NMR: ($CDCl_3$, 100 MHz) 24.7, 26.0, 28.4, 29.0, 30.5, 32.2, 47.2, 50.9, 51.8, 52.7, 60.8, 66.2, 67.0, 79.6, 117.6, 127.3, 128.1, 128.2, 128.3, 128.4, 128.5, 128.5, 129.1, 136.0, 136.9, 137.8, 155.1, 171.3, 171.4, 171.8, 172.9.

*1.10. L-histidinyl(OBn)-L-prolyl-L-glutamyl dibenzyl ester (**12**)*

To a round bottom flask containing Boc-L-histidinyl(OBn)-L-prolyl-L-glutamyl dibenzyl ester **11** (5.4 g, 7.19 mmol) was added 10 mL of TFA/DCM (1:1) and stirred for 1 h at RT. The organic solvents were evaporated and the resultant mixture was purified by column chromatography using DCM/MeOH (96:4) as eluent to obtain compound **12** (Figure S10) as a pale yellow liquid (4.3 g, 93%).

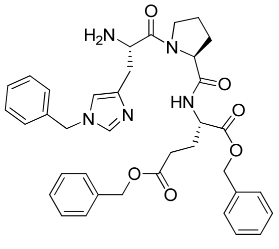

(**12**)

Figure S10. L-Histidinyl(OBn)-L-prolyl-L-glutamyl dibenzyl ester.

^{1}H NMR: ($CDCl_3$, 400 MHz) 1.84–1.86 (m, 3H); 2.05–2.25 (m, 3H); 2.42 (t, 2H, *J* = 7.44 Hz); 3.15–3.56 (m, 3H); 3.57–3.60 (m, 1H); 4.50–4.51 (m, 3H); 5.00–5.12 (m, 4H); 5.00 (s, 2H); 5.02–5.12 (m, 4H); 7.21–7.34 (m, 15H); 8.04 (s, 1H); 8.56–8.58 (d, 1H, *J* = 6.12 Hz). [13] C NMR: ($CDCl_3$, 100 MHz) 24.9, 26.3, 29.2, 30.3, 31.0, 47.6, 51.2, 52.1, 52.5, 60.7, 66.4, 67.0, 115.2, 118.1, 121.6, 128.1, 128.1, 128.4, 128.5, 129.3, 133.7, 135.4, 135.8, 162.0, 167.2, 171.4, 172.9.

*1.11. Boc-glycyl-L-histidinyl(OBn)-L-prolyl-L-glutamyl Dibenzyl Ester (**13**)*

To a solution of L-histidinyl(OBn)-L-prolyl-L-glutamyl dibenzyl ester **12** (4.3 g, 6.6 mmol) and triethylamine (1336 mg, 13.3 mmol) in dichloromethane (20 mL) in a round bottom flask was added Boc-glycine (1156 mg, 6.6 mmol), DCC (1362 mg, 6.6 mmol) and HOBt (891 mg, 6.6 mmol) at 0 °C. The reaction mixture was stirred for 2 h at 0 °C and 17 h at RT. The resultant white mixture was filtered to remove 1, 3-dicyclohexylurea. The filtrate was evaporated and the residue was dissolved in ethyl acetate. The organic layer was washed successively with 5% aq. citric acid solution, water, 5% aq. $NaHCO_3$ solution and water. The organic layer was dried over Na_2SO_4 and concentrated. The resultant mixture was purified by column chromatography using DCM/MeOH (96:4) as eluent to obtain compound **13** (Figure S11) as a white solid (4.4 g, 83%).

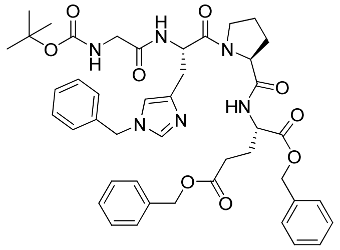

(**13**)

Figure S11. Boc-glycyl-L-histidinyl(OBn)-L-prolyl-L-glutamyl dibenzyl ester.

^{1}H NMR: ($CDCl_3$, 400 MHz) 1.42 (s, 9H); 1.66–1.76 (m, 2H); 1.97–2.08 (m, 2H); 2.16–3.49 (m, 4H); 2.84–2.98 (m, 3H); 3.44 (q, 1H); 3.77–3.78 (d, 2H, *J* = 4.96 Hz); 4.48 (q, 1H); 4.56 (dd, 1H, *J* = 2.76 Hz, *J* = 8.44 Hz); 4.73–4.78 (m, 1H); 4.92 (s, 2H); 4.95–5.12 (m, 4H); 5.23 (s, 1H); 6.73 (s, 1H); 7.09–7.11 (m, 2H); 7.23–7.34 (m, 15H); 9.24–9.26 (d, 1H, *J* = 7.48 Hz). [13] C NMR: ($CDCl_3$, 100 MHz) 24.6, 26.1, 28.3, 29.1, 30.5, 31.5, 44.0, 47.4, 50.9, 51.5, 51.8, 60.8, 66.2, 66.9, 80.1, 117.8, 127.4, 128.1, 128.2, 128.2, 128.4, 128.5, 128.5, 129.0, 135.7, 135.8, 136.0, 136.9, 137.4, 155.9, 168.7, 170.8, 171.4, 171.8, 172.8.

*1.12. Boc-glycyl-L-histidinyl(OBn)-L-prolyl-L-glutamic Acid-γ(OBn) (**14**)*

To a solution of B. *subtilis* protease (Sigma type–VIII) (50 mg) in 32 mL of 0.1 M, pH 7, phosphate buffer was added drop by drop a solution of Boc-glycyl-L-histidinyl(OBn)-L-prolyl-L-glutamyl dibenzyl ester **13** (4.4 g, 5.44 mmol) in acetone (8 mL). The reaction mixture was stirred at 35 °C overnight. Then the solution was basified to pH 8 to remove unchanged ester by ethyl acetate. The aqueous layer was acidified to pH 2 and centrifuged to remove enzyme. The resultant mixture was extracted with ethyl acetate and dried over Na_2SO_4. The organic portion was evaporated and purified by column

chromatography using DCM/MeOH (96:4) as eluent to obtain compound **14** (Figure S12) as a white solid (3.36 g, 86%).

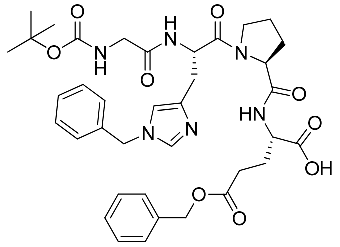

(**14**)

Figure S12. Boc-glycyl-L-histidinyl(OBn)-L-prolyl-L-glutamic acid-γ(OBn).

^{1}H NMR: (CD_3OD, 400 MHz) 1.42 (s, 9H); 1.90–2.01 (m, 4H); 2.20–2.29 (m, 2H); 2.47–2.55 (m, 2H); 2.98 (dd, 1H, *J* = 5.6 Hz, *J* = 15.08 Hz); 3.17 (dd, 1H, *J* = 6.8 Hz, *J* = 15.04 Hz); 3.45–3.52 (m, 1H); 3.62 (s, 2H); 4.44 (q, 1H); 4.50 (q, 1H); 4.93 (s, 1H); 5.09 (q, 2H); 5.34 (s, 2H); 7.23–7.34 (m, 15H); 7.40 (s, 1H); 8.88 (s, 1H). ^{13}C NMR: (CD_3OD, 100 MHz) 24.6, 26.4, 26.6, 27.3, 29.3, 29.9, 43.0, 50.0, 51.5, 52.5, 60.2, 63.8, 66.0, 79.4, 110.0, 117.2, 120.8, 127.0, 127.8, 127.8, 128.0, 128.2, 128.3, 129.0, 129.0, 134.0, 134.5, 136.1, 141.3, 168.5, 170.7, 172.8, 173.1, 173.4.

1.13. Cyclo(glycyl-L-histidinyl(OBn)-L-prolyl-L-glutamyl(OBn) (4)

Similar procedure as for the synthesis of compound **3** was applied to obtain compound **4** (Figure S13) as a white solid with an overall yield of 52%.

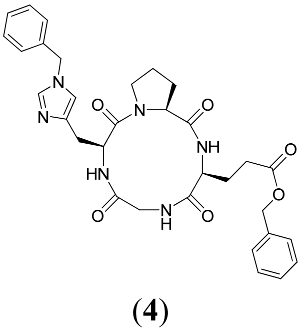

(**4**)

Figure S13. Cyclo(glycyl-L-histidinyl(OBn)-L-prolyl-L-glutamyl(OBn).

^{1}H NMR: (CD_3OD & $CDCl_3$, 400 MHz) 143–1.63 (m, 3H); 1.92 (s, 2H); 2.02–2.30 (m, 3H); 2.75–2.89 (m, 1H); 2.96–2.99 (m, 1H); 3.35–3.48 (m, 3H); 3.71 (s, 2H); 4.68 (s, 1H); 4.73–4.75 (m, 1H); 4.86–4.93 (m, 2H); 5.07–5.17 (m, 2H); 6.92 (s, 1H); 7.14–7.26 (m, 10H); 8.49 (s, 1H). ^{13}C NMR: (CD_3OD & $CDCl_3$, 100 MHz) 25.4, 25.9, 30.3, 33.6, 35.8, 49.3, 51.5, 51.6, 56.7, 56.9, 65.6, 70.3, 122.5, 124.2, 132.0, 132.1, 132.1, 132.2, 132.3, 132.4, 133.2, 133.3, 135.6, 137.0, 139.7, 173.4, 174.3, 176.1, 176.7, 178.3. MALDI-TOF: $[M + H]^+$ calculated *m/z* 601.277, obtained *m/z* 601.383.

*1.14. Cyclo(glycyl-L-histidinyl-L-prolyl-L-glutamyl) (**2**)*

Cyclo(glycyl-L-histidinyl(OBn)-L-prolyl-L-glutamyl(OBn) **4** (100 mg, 0.17 mmol) was dissolved in 5 mL of DMF and 10% palladium hydroxide on carbon was added under nitrogen. The vessel was purged three times with nitrogen and then the bottle was shaken at 50 lbs per sq inch hydrogen pressure in a parr shaker for 8 h. The catalyst was removed by filtration and the filtrate was evaporated to obtain compound **2** (Figure S14) as a white powder (77 mg, 95%) $[\alpha]^{21}_{D} = -117.75$ (C = 0.1, H_2O).

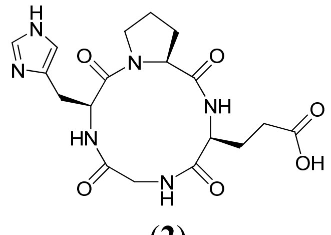

(**2**)

Figure S14. Cyclo(glycyl-L-histidinyl-L-prolyl-L-glutamyl).

^{1}H NMR: (D_2O, 400 MHz) 1.56–2.02 (m, 5H); 2.16 (s, 3H); 2.83–2.93 (m, 1H); 3.10–3.20 (m, 1H); 3.39–3.58 (m, 2H); 3.63–3.75 (m, 2H); 3.80–3.95 (m, 1H); 4.08 (dd, 1H, *J* = 8.56 Hz, *J* = 40.16 Hz); 4.88–4.98 (m, 1H); 7.14 (s, 1H); 8.44 (s,1H). ^{13}C NMR: (D_2O, 100 MHz) 21.3, 24.2, 26.0, 30.8, 31.5, 46.0, 48.0, 50.3, 51.8, 59.5, 117.4, 129.0, 133.0, 168.8, 170.6, 171.1, 173.8, 175.6. IR (cm^{-1}): 3272.12, 1668.93, 1541.19, 1443.98, 1346.04, 1205.54, 1135.58, 981.93, 838.79, 799.63, 749.84, 722.56, 670.75, 629.16, 604.80, 477.38. MALDI-TOF: $[M + H]^+$ calculated *m/z* 421.183, obtained *m/z* 421.296. HRMS (ESI): $[M + H]^+$ calculated: *m/z* 421.1830, obtained: *m/z* 421.1841.

2. Spectra

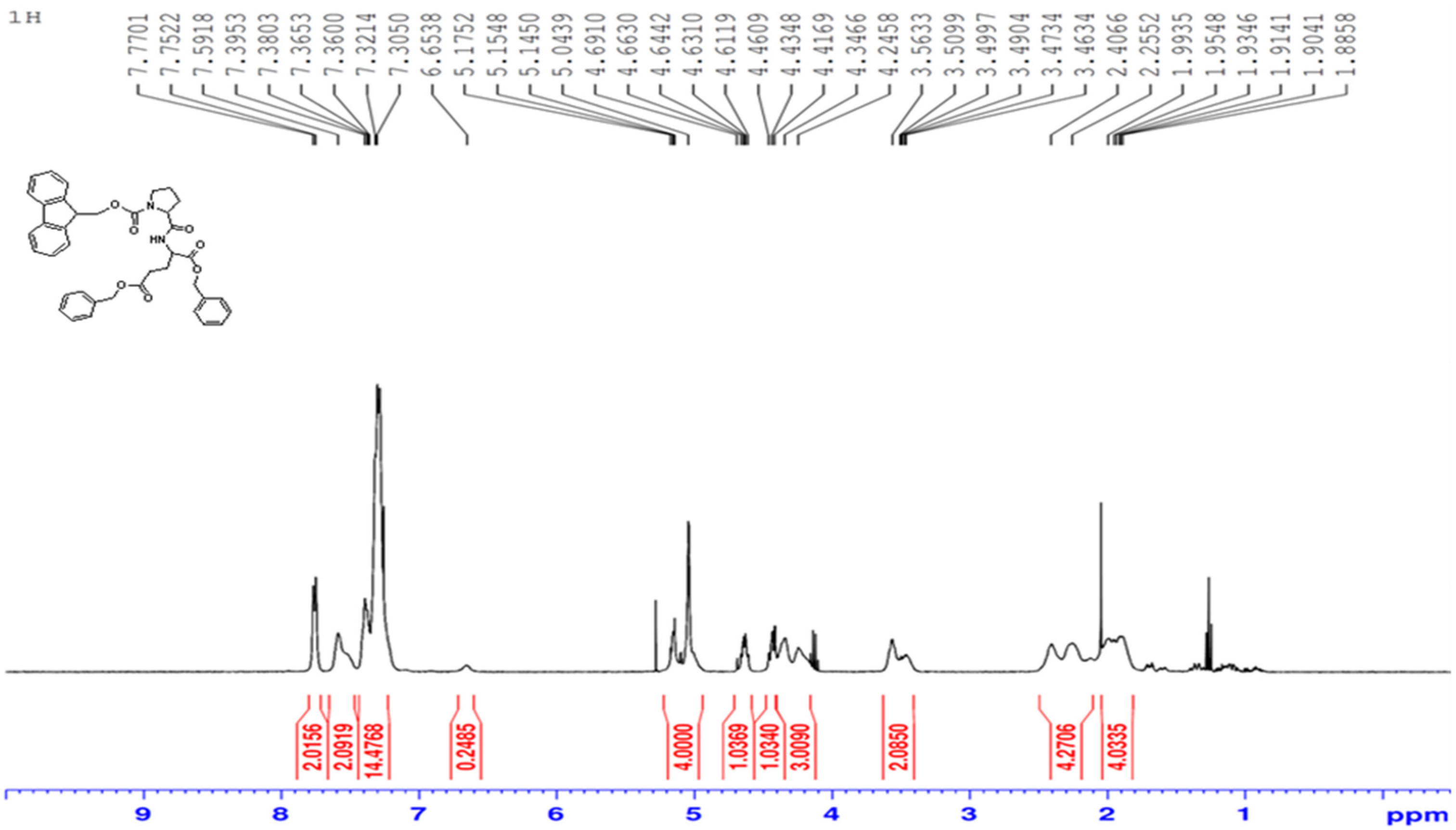

Figure S15. ^{1}H NMR: ($CDCl_3$, 400 MHz) of Fmoc-L-prolyl-L-glutamyl dibenzyl ester (compound **5**).

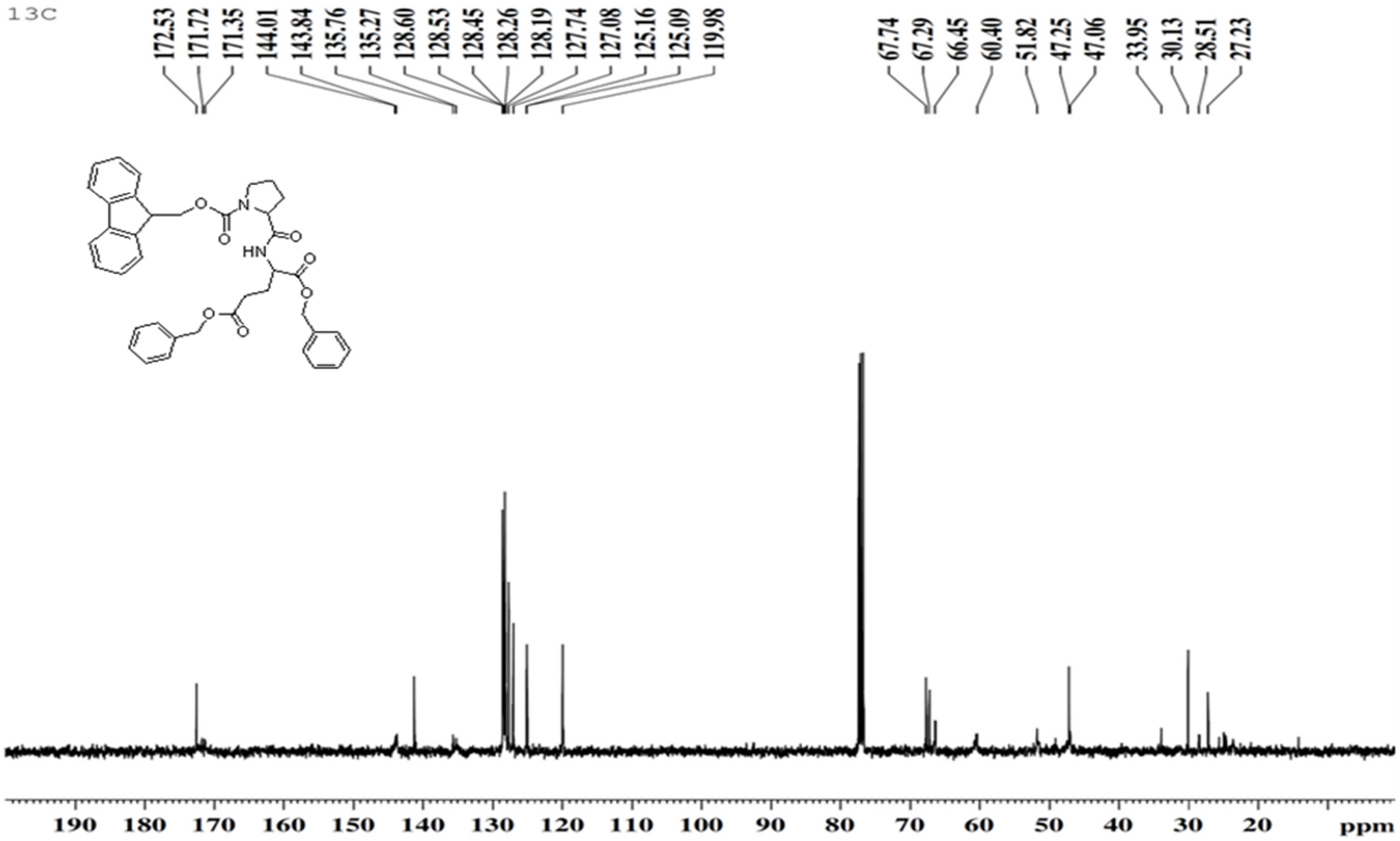

Figure S16. ^{13}C NMR: ($CDCl_3$, 100 MHz) of Fmoc-L-prolyl-L-glutamyl dibenzyl ester (compound **5**).

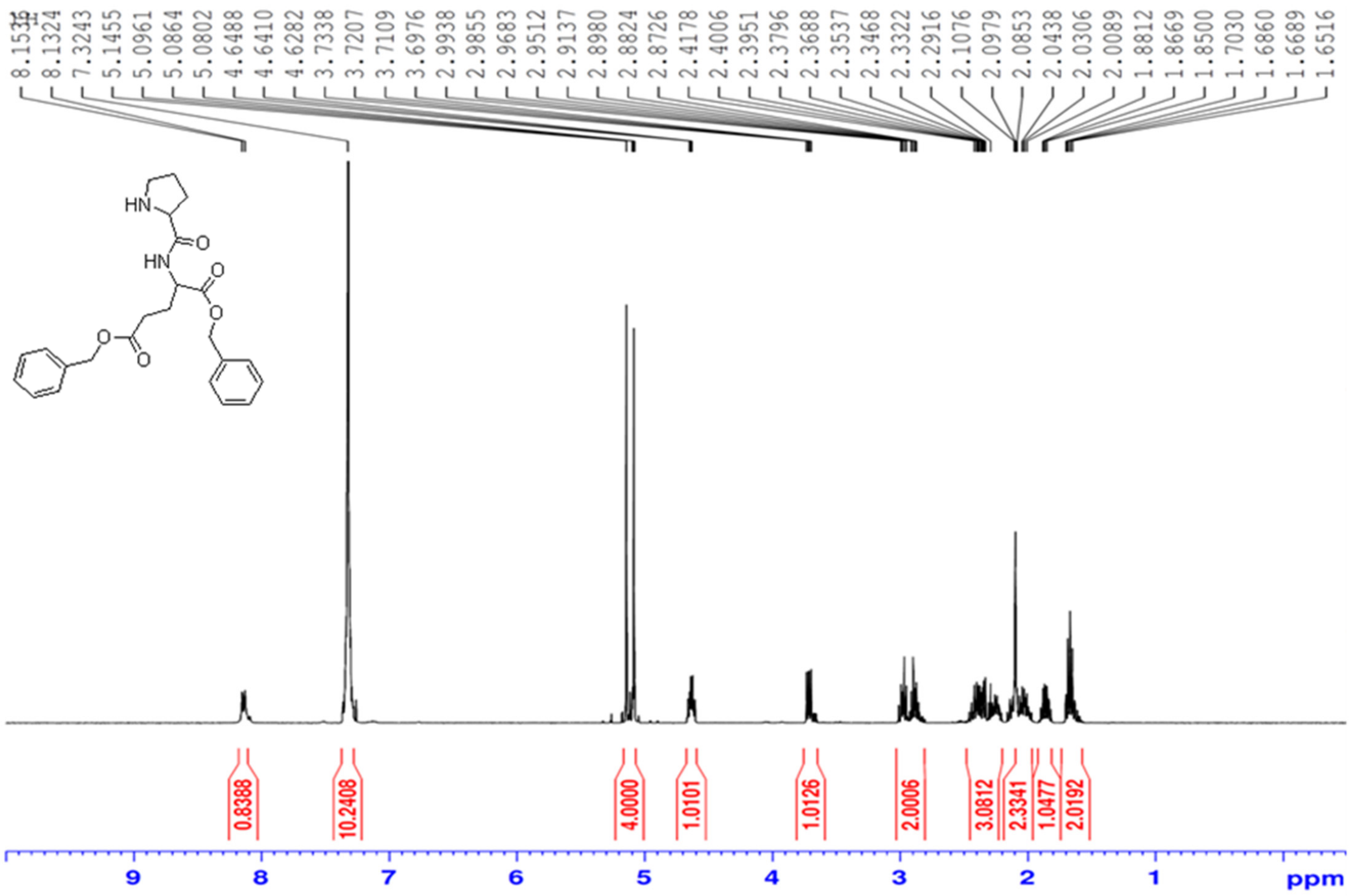

Figure S17. ^{1}H NMR: ($CDCl_3$, 400 MHz) of L-Prolyl-L-glutamyl dibenzyl ester (compound **6**).

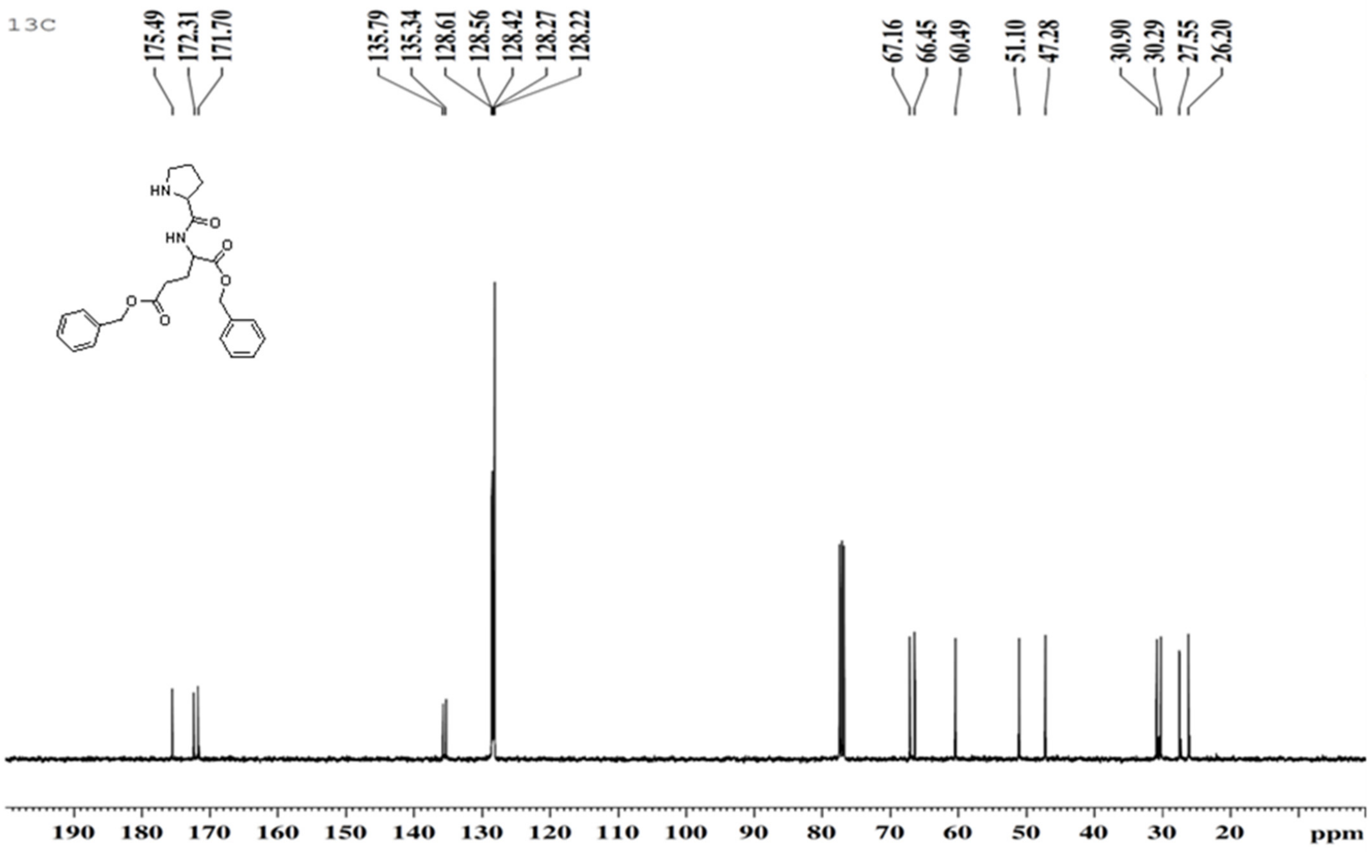

Figure S18. ^{13}C NMR: ($CDCl_3$, 100 MHz) of L-Prolyl-L-glutamyl dibenzyl ester (compound **6**).

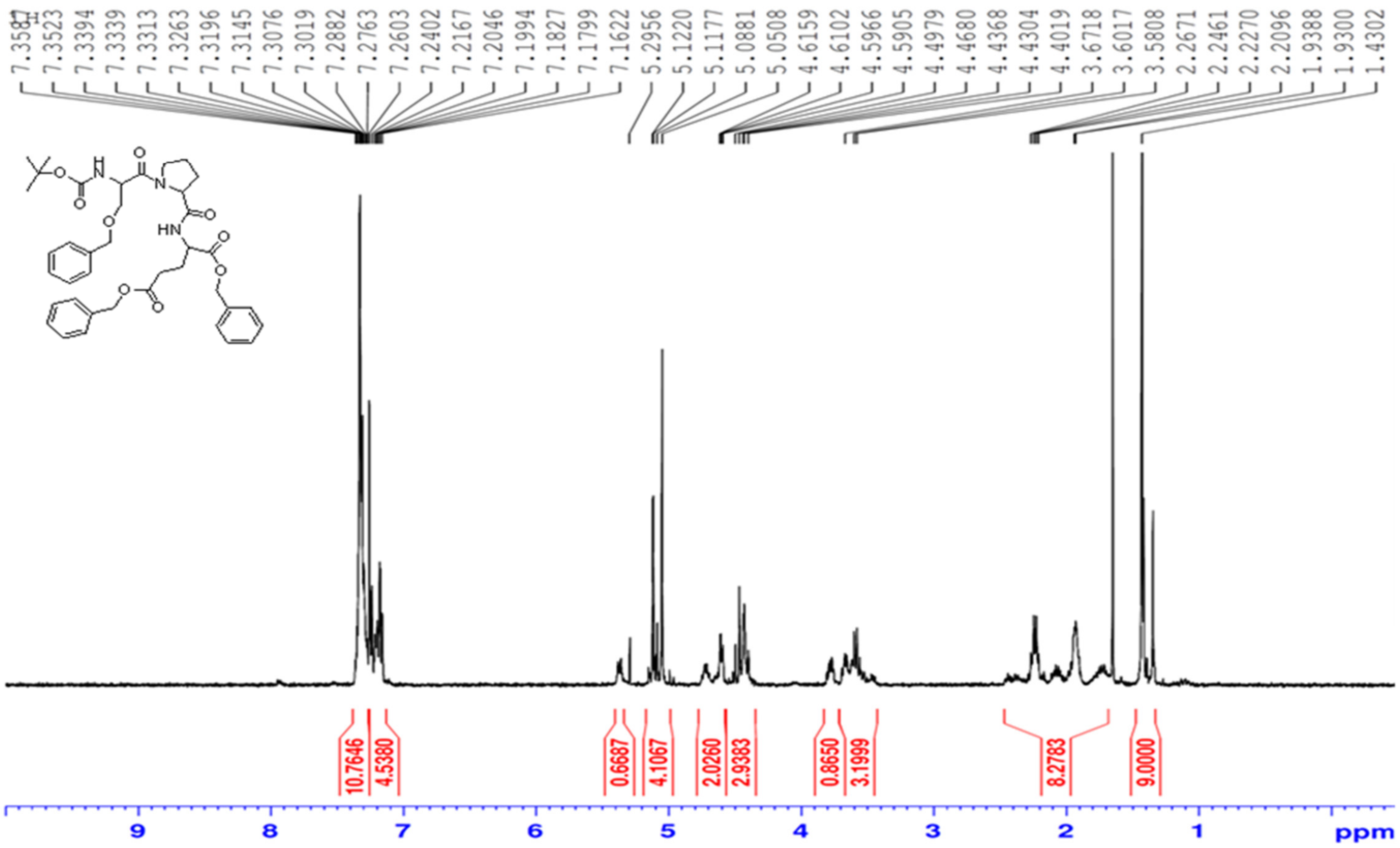

Figure S19. ^{1}H NMR: ($CDCl_3$, 400 MHz) of Boc-L-seryl(OBn)-L-prolyl-L-glutamate dibenzyl ester (compound **7**).

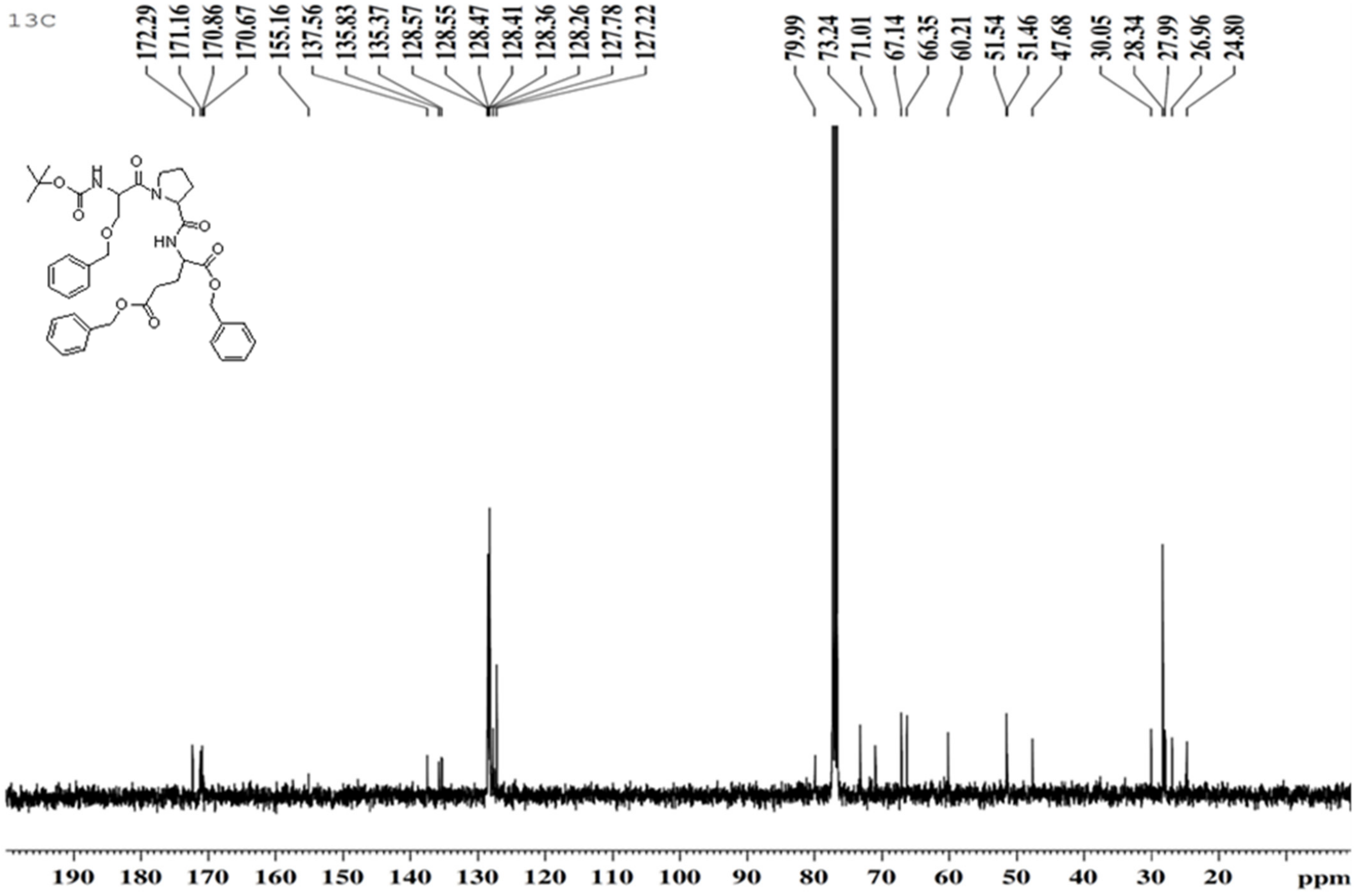

Figure S20. ^{13}C NMR: ($CDCl_3$, 100 MHz) of Boc-L-seryl(OBn)-L-prolyl-L-glutamate dibenzyl ester (compound **7**).

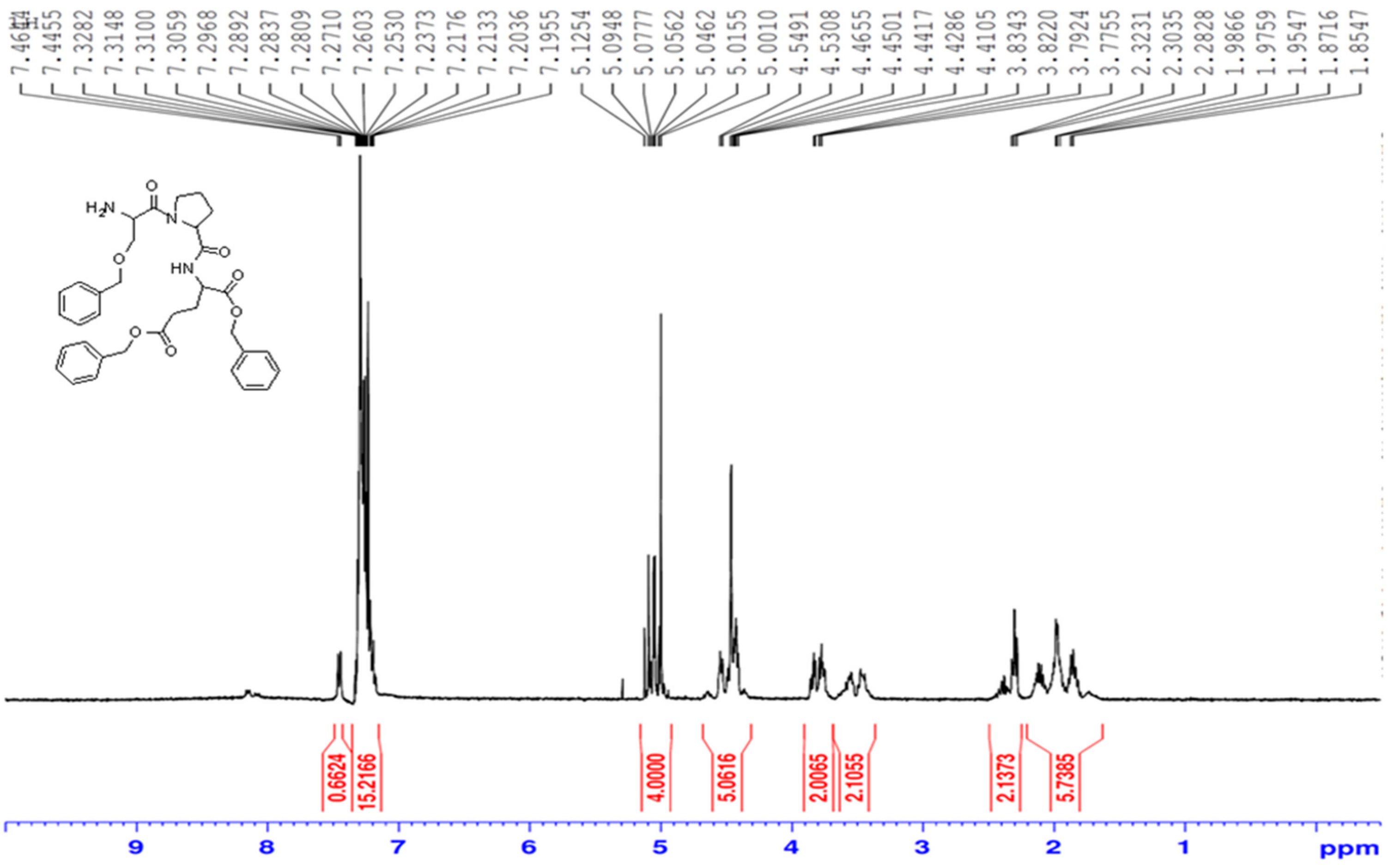

Figure S21. ^{1}H NMR: ($CDCl_3$, 400 MHz) of L-Seryl(OBn)-L-prolyl-L-glutamate dibenzyl ester (compound **8**).

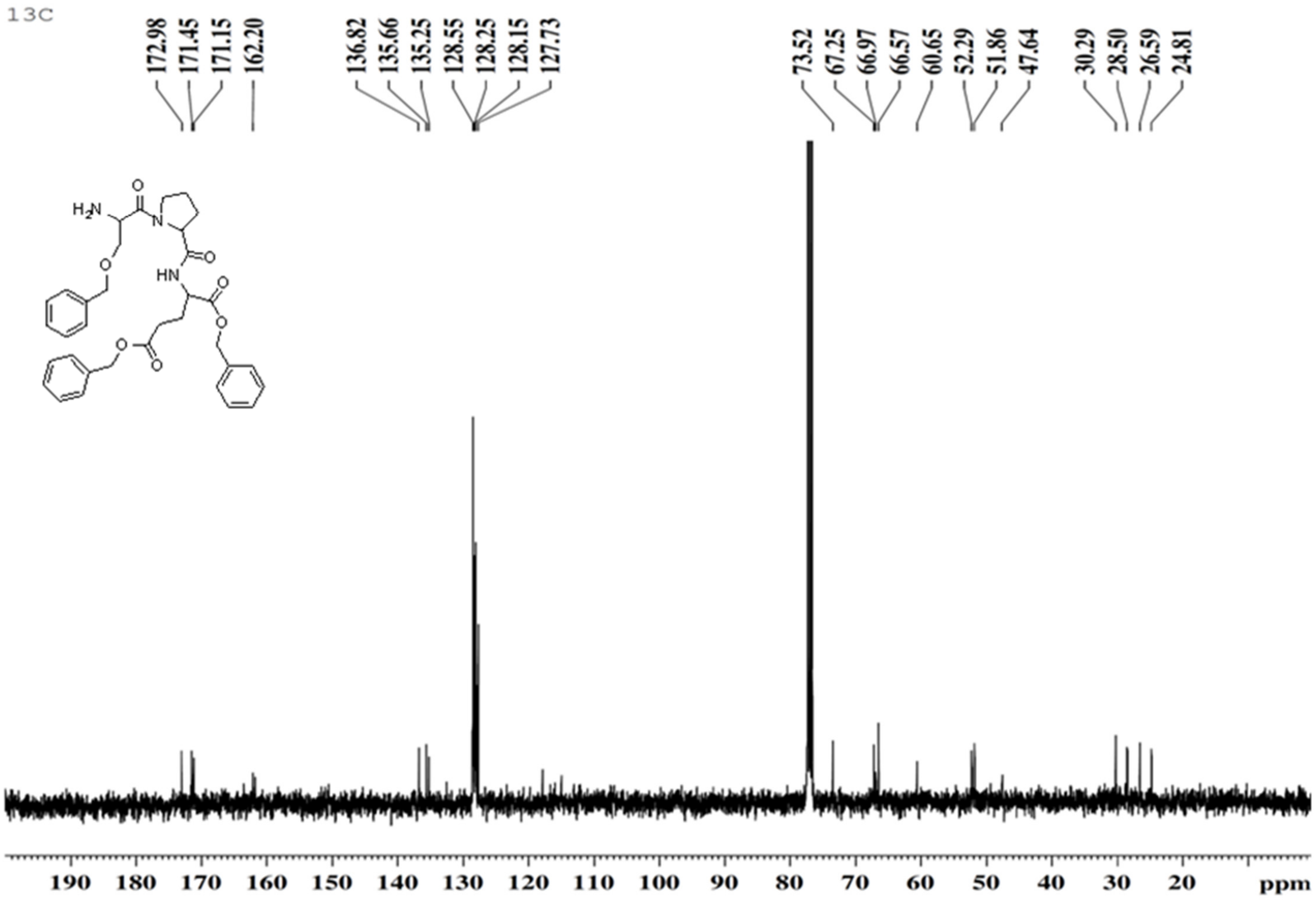

Figure S22. ^{13}C NMR: ($CDCl_3$, 100 MHz) of L-Seryl(OBn)-L-prolyl-L-glutamate dibenzyl ester (compound **8**).

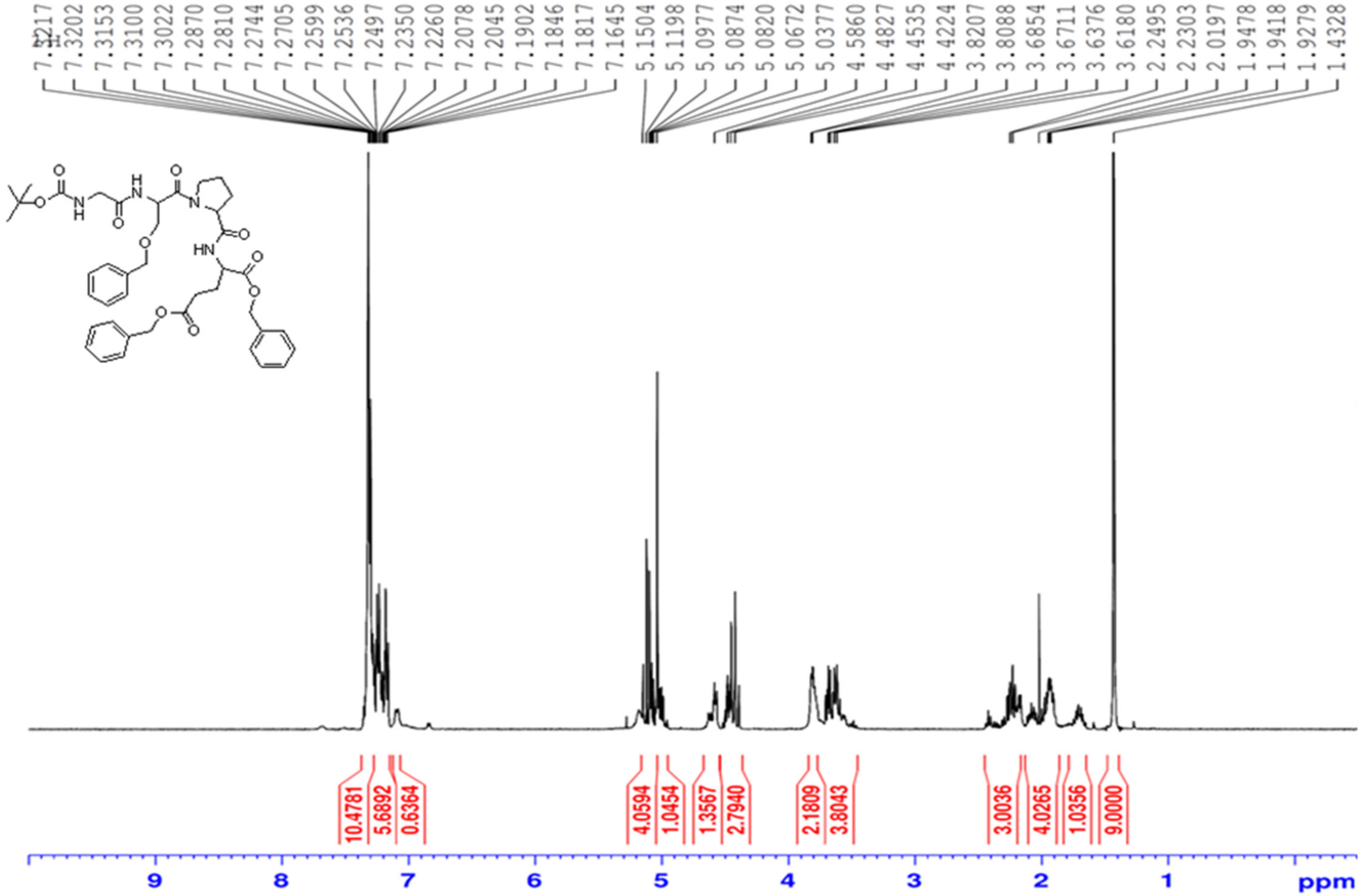

Figure S23. ^{1}H NMR: ($CDCl_3$, 400 MHz) of Boc-glycyl-L-seryl(OBn)-L-prolyl-L-glutamate dibenzyl ester (compound **9**).

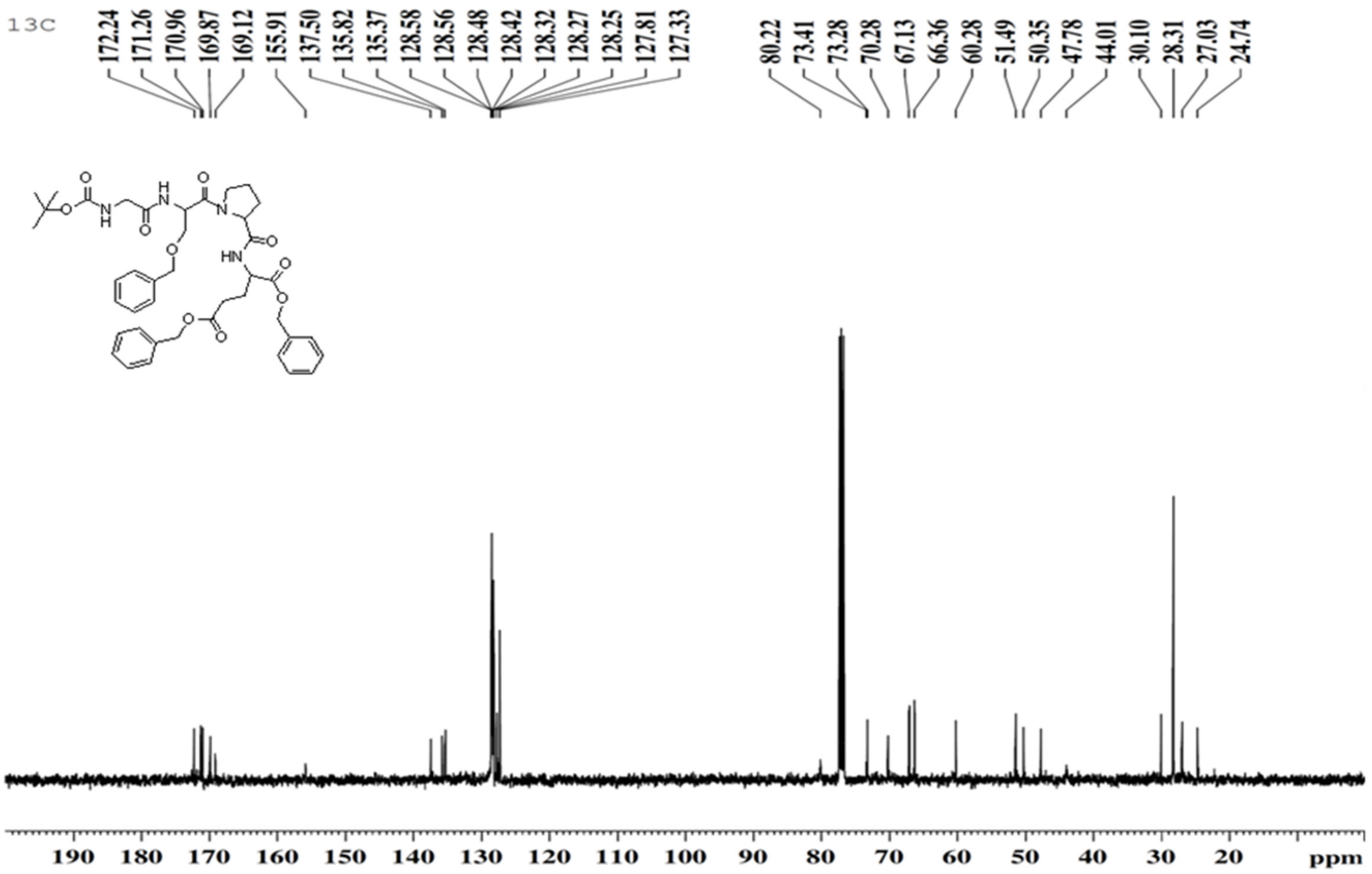

Figure S24. ^{13}C NMR: ($CDCl_3$, 100 MHz) of Boc-glycyl-L-seryl(OBn)-L-prolyl-L-glutamate dibenzyl ester (compound **9**).

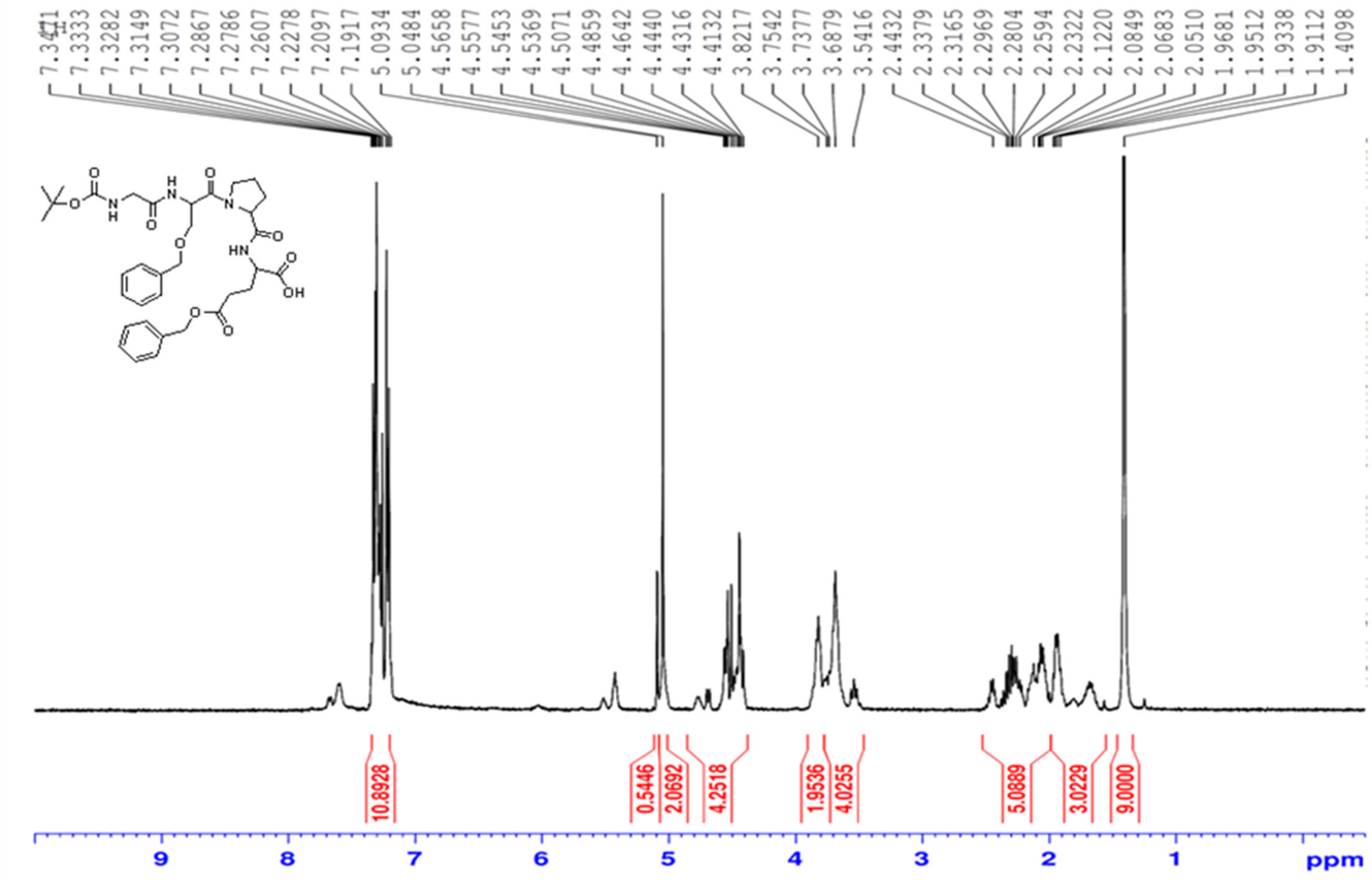

Figure S25. ^{1}H NMR: ($CDCl_3$,400 MHz) Boc-glycyl-L-sery(OBn)-L-prolyl-L-glutamic acid-α-COOH -γ-OBn (compound **10**).

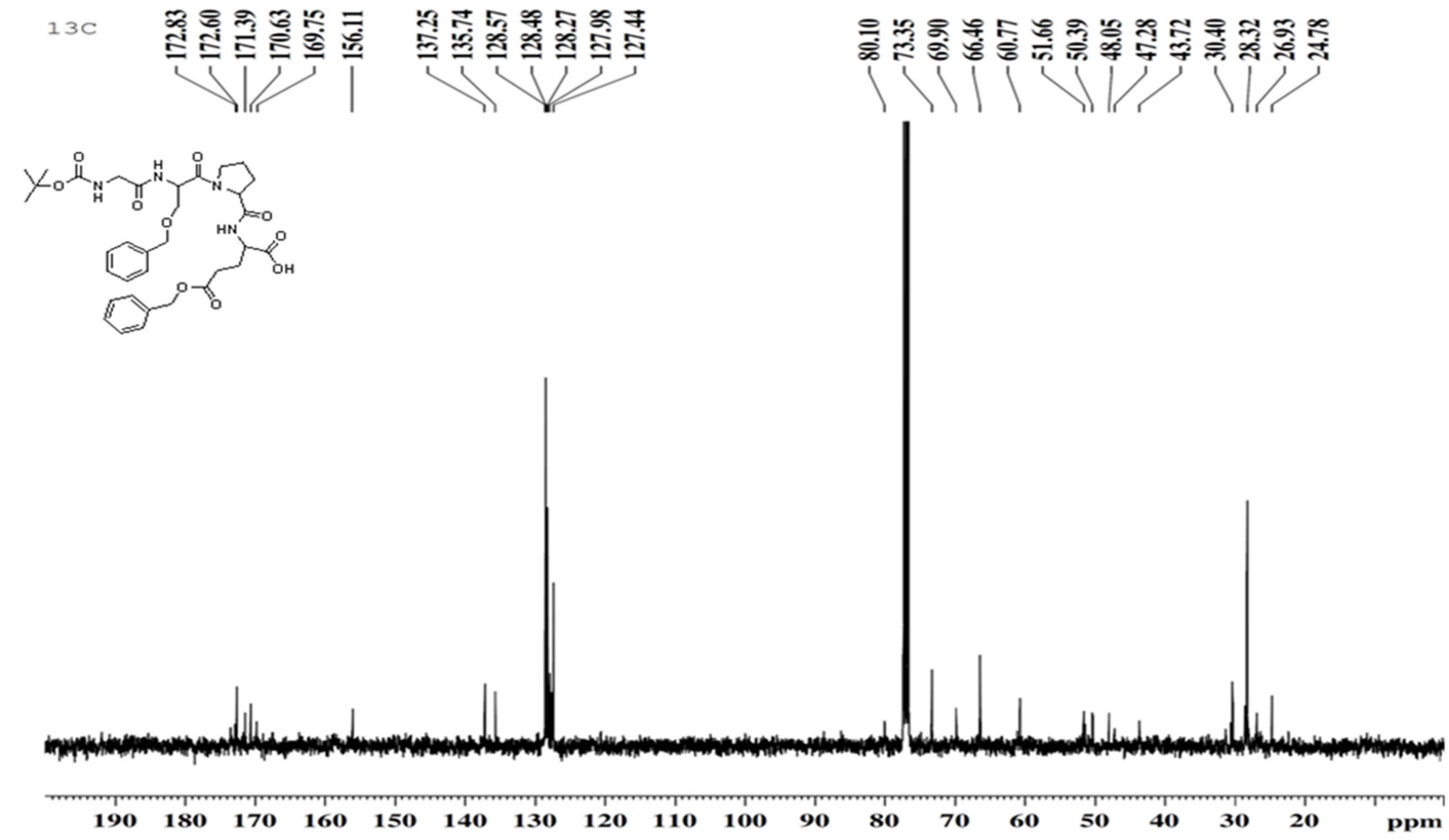

Figure S26. ^{13}C NMR:(CDCl3,100MHz) Boc-glycyl-L-sery(OBn)-L-prolyl-L-glutamic acid-α-COOH-γ-OBn (compound **10**).

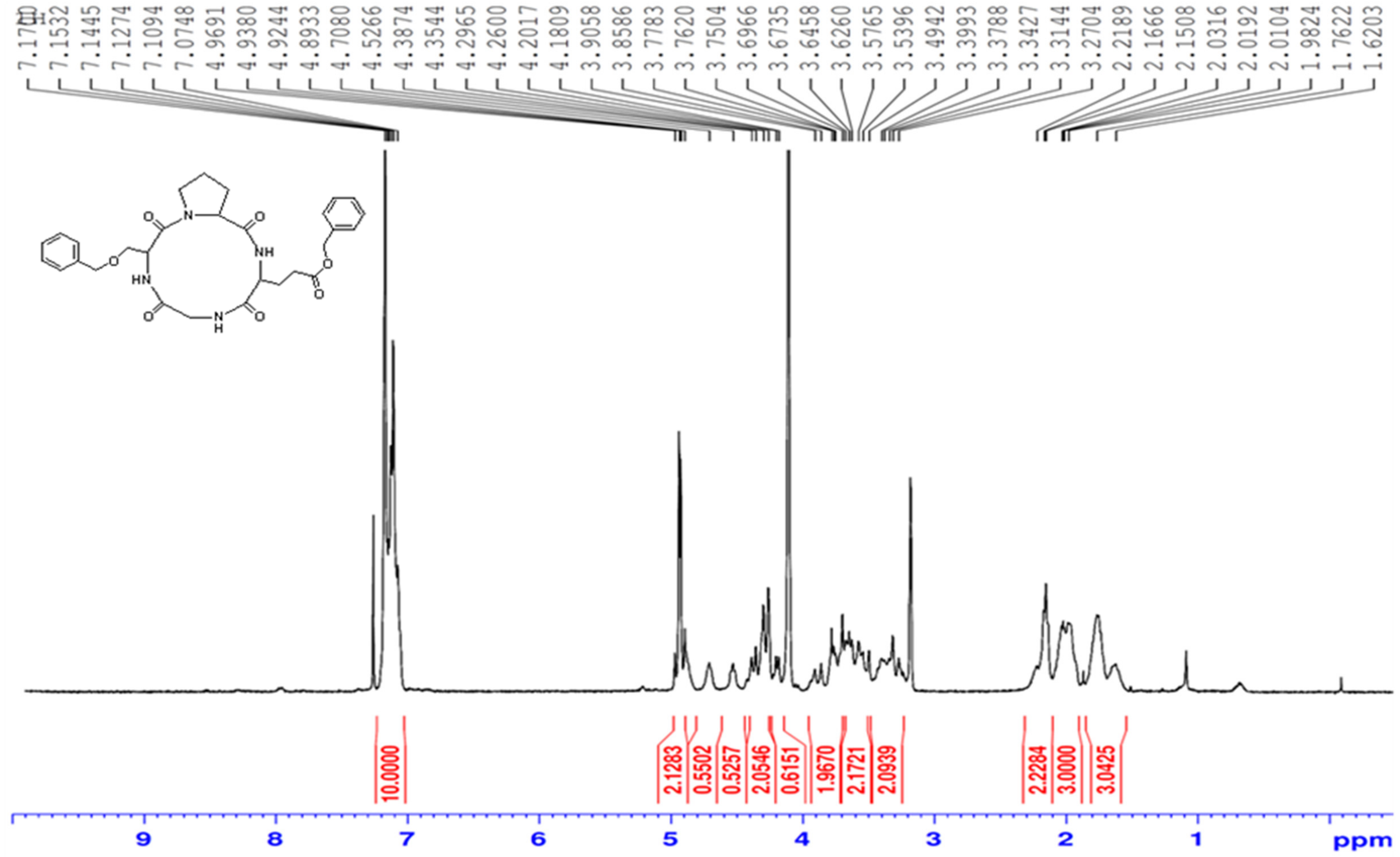

Figure S27. ^{1}H NMR: ($CDCl_3$ & CD_3OD,400 MHz) *Cyclo*(glycyl-L-seryl(OBn)-L-prolyl-L-glutamyl(OBn) (compound **3**).

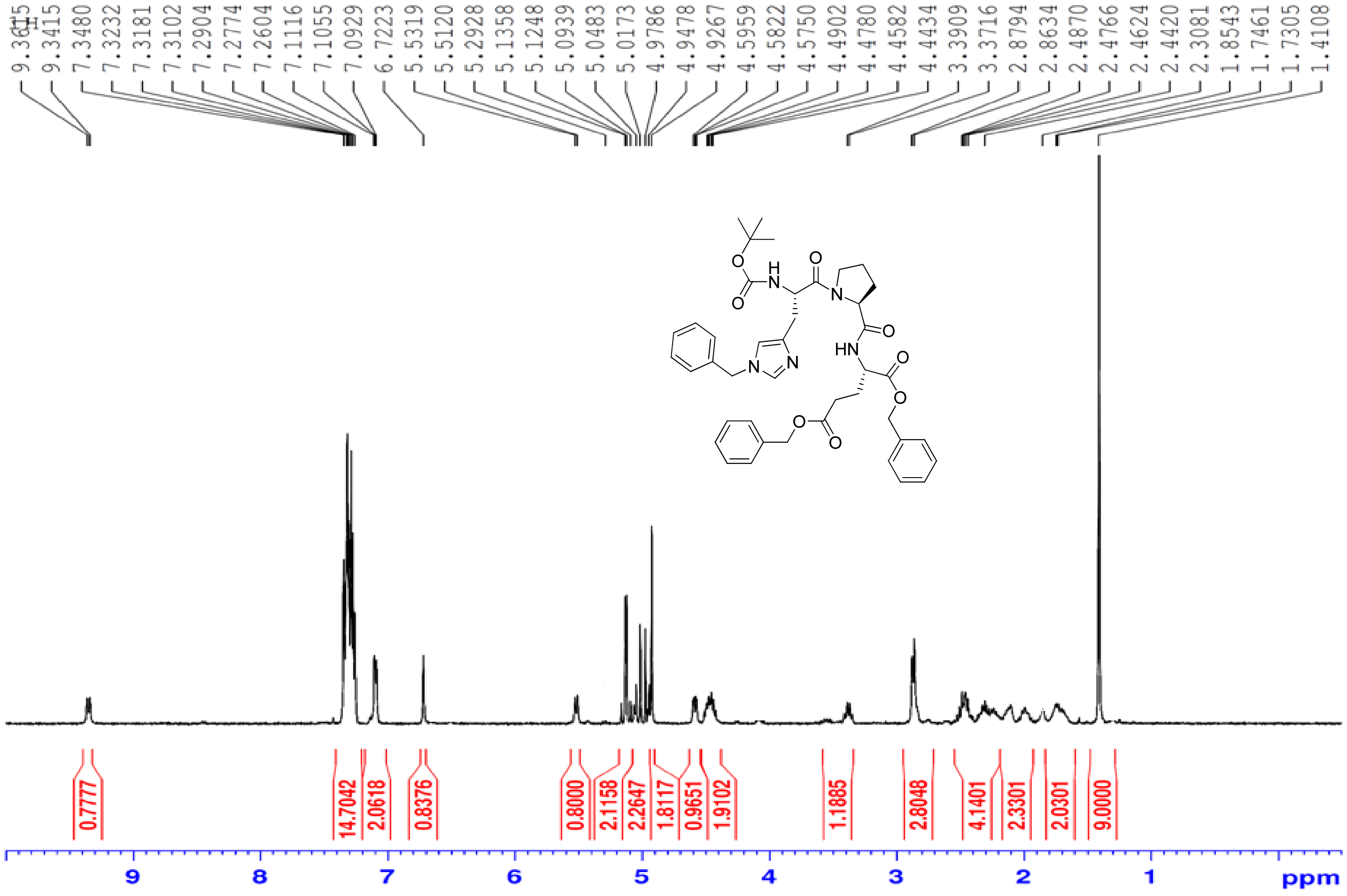

Figure S28. ^{1}H NMR: ($CDCl_3$, 400 MHz) of Boc-L-histidinyl(OBn)-L-prolyl-L-glutamyl dibenzyl ester (compound **11**).

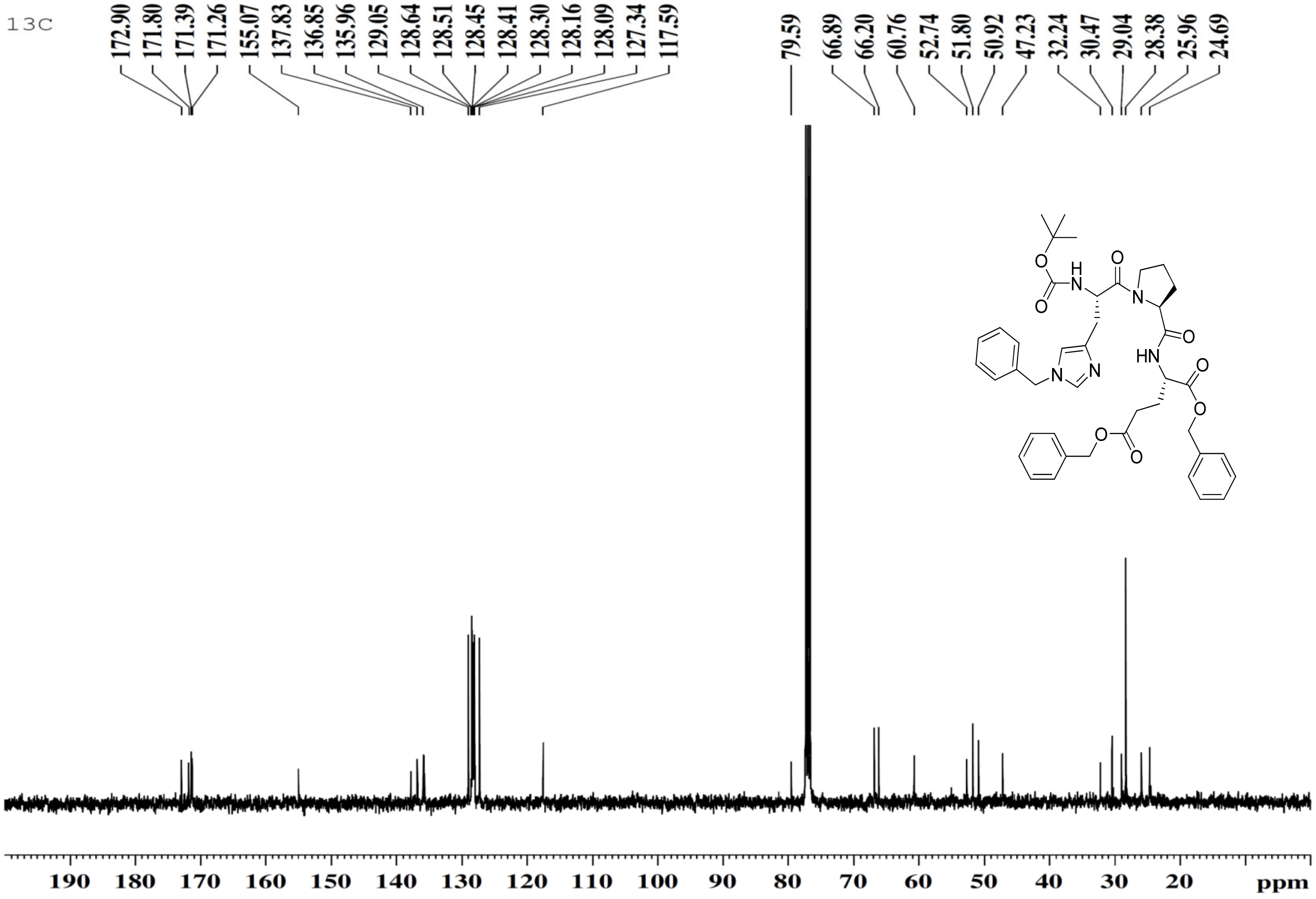

Figure S29. ^{13}C NMR: ($CDCl_3$, 100 MHz) of Boc-L-histidinyl(OBn)-L-prolyl-L-glutamyl dibenzyl ester (compound **11**).

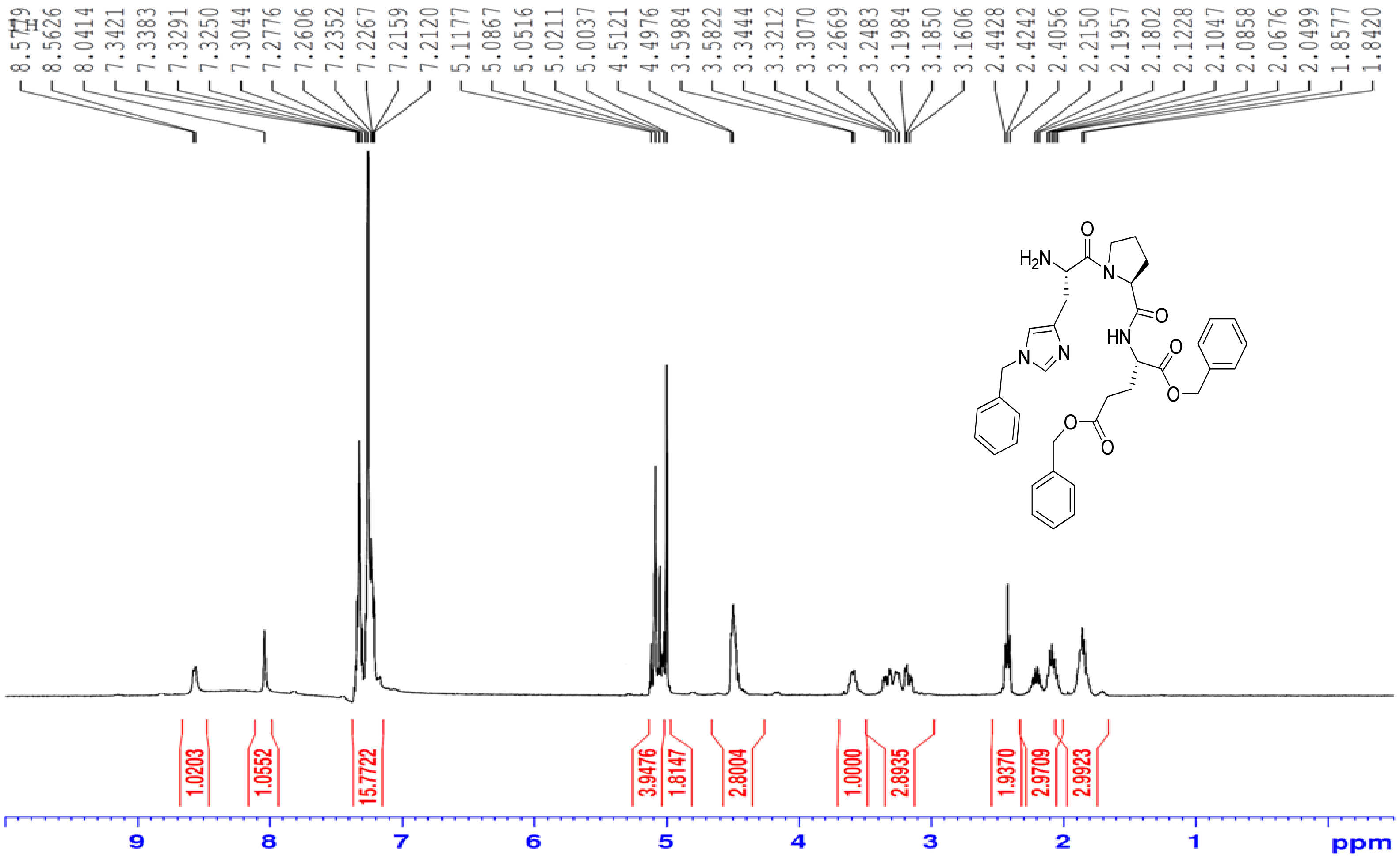

Figure S30. ^{1}H NMR: ($CDCl_3$, 400 MHz) of L-histidinyl(OBn)-L-prolyl-L-glutamyl dibenzyl ester (compound **12**).

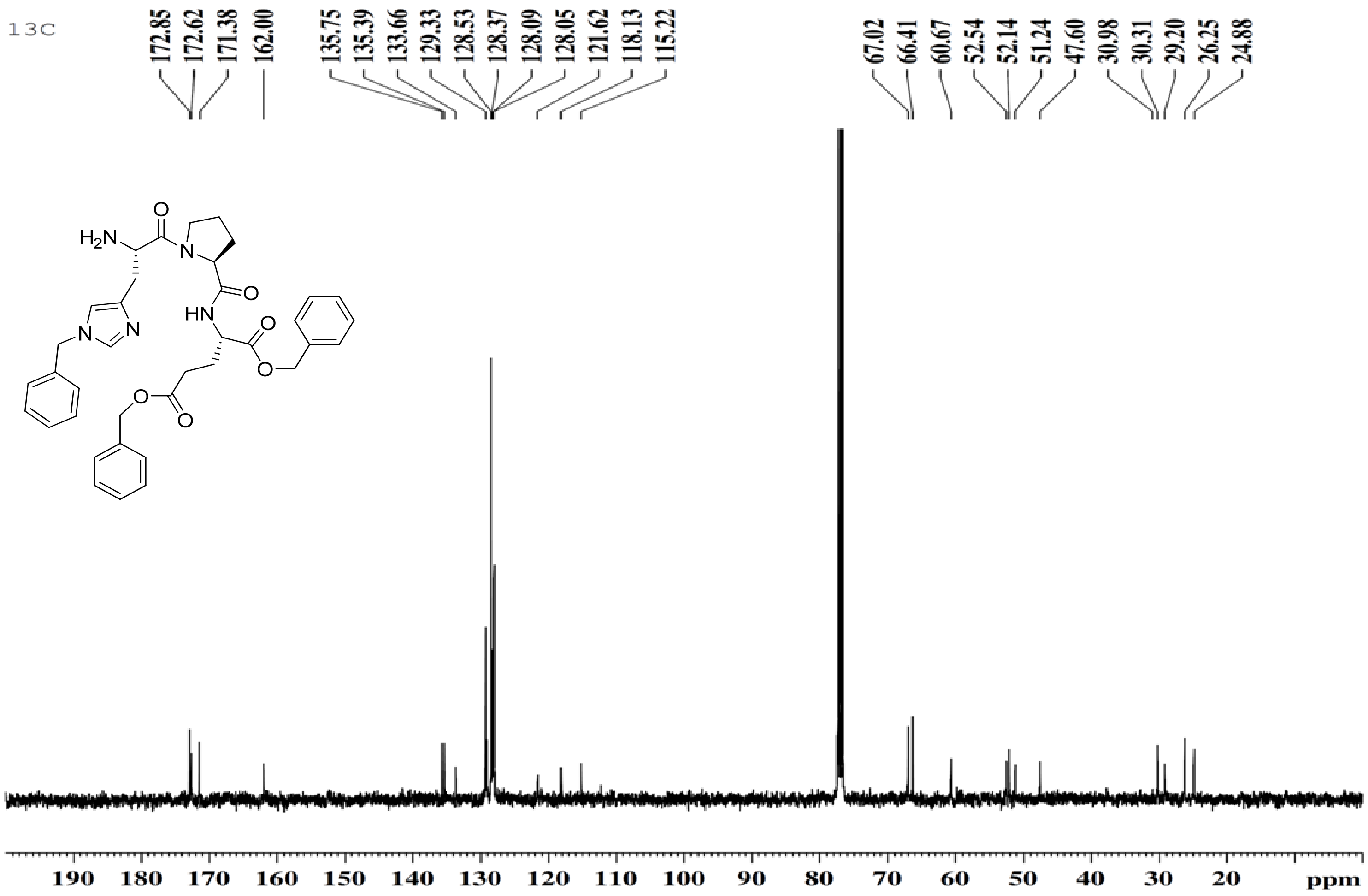

Figure S31. ^{13}C NMR: ($CDCl_3$, 100 MHz) of L-histidinyl(OBn)-L-prolyl-L-glutamyl dibenzyl ester (compound **12**).

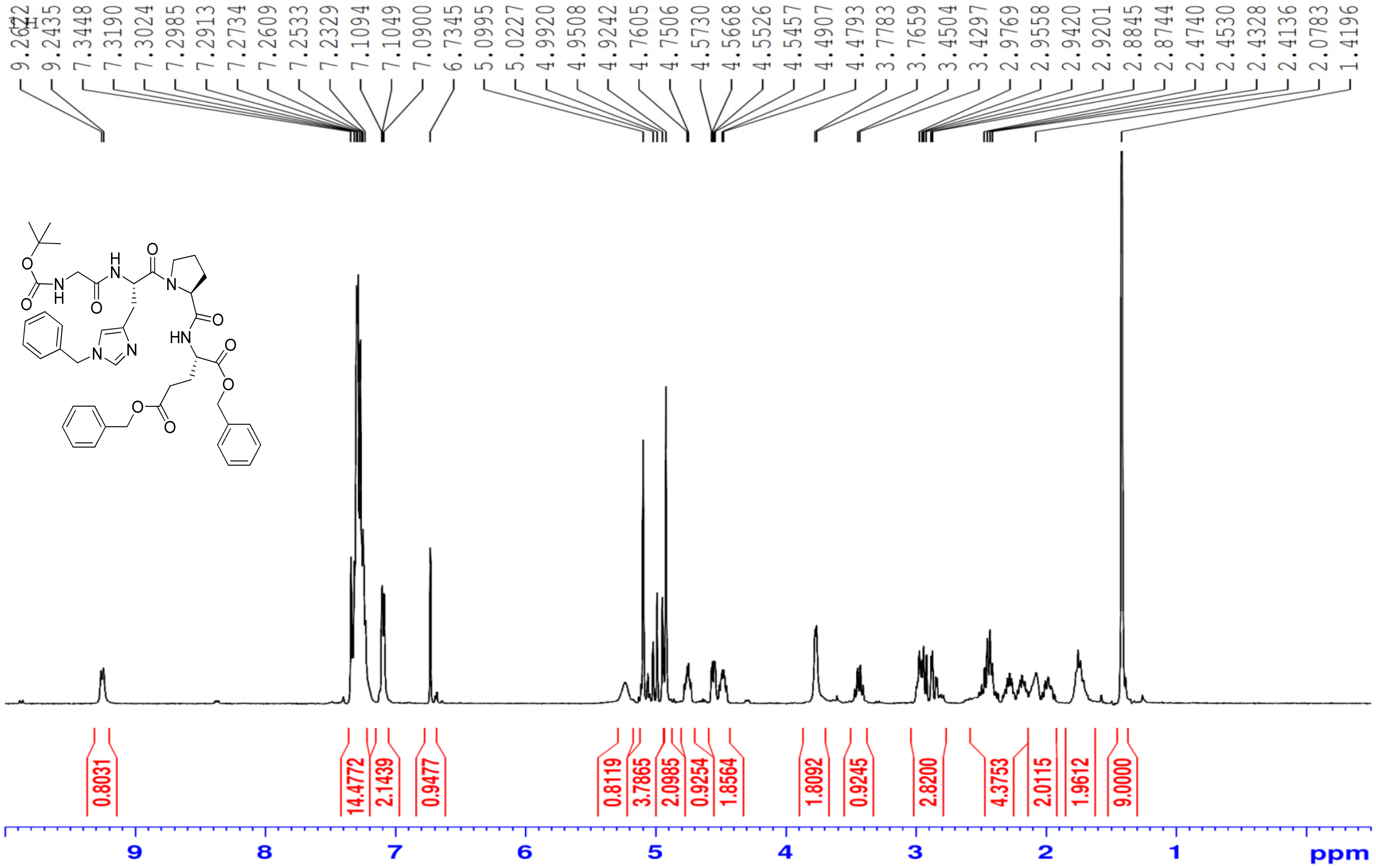

Figure S32. ^{1}H NMR: ($CDCl_3$, 400 MHz) of Boc-glycyl-L-histidinyl(OBn)-L-prolyl-l-glutamyl dibenzyl ester (compound **13**).

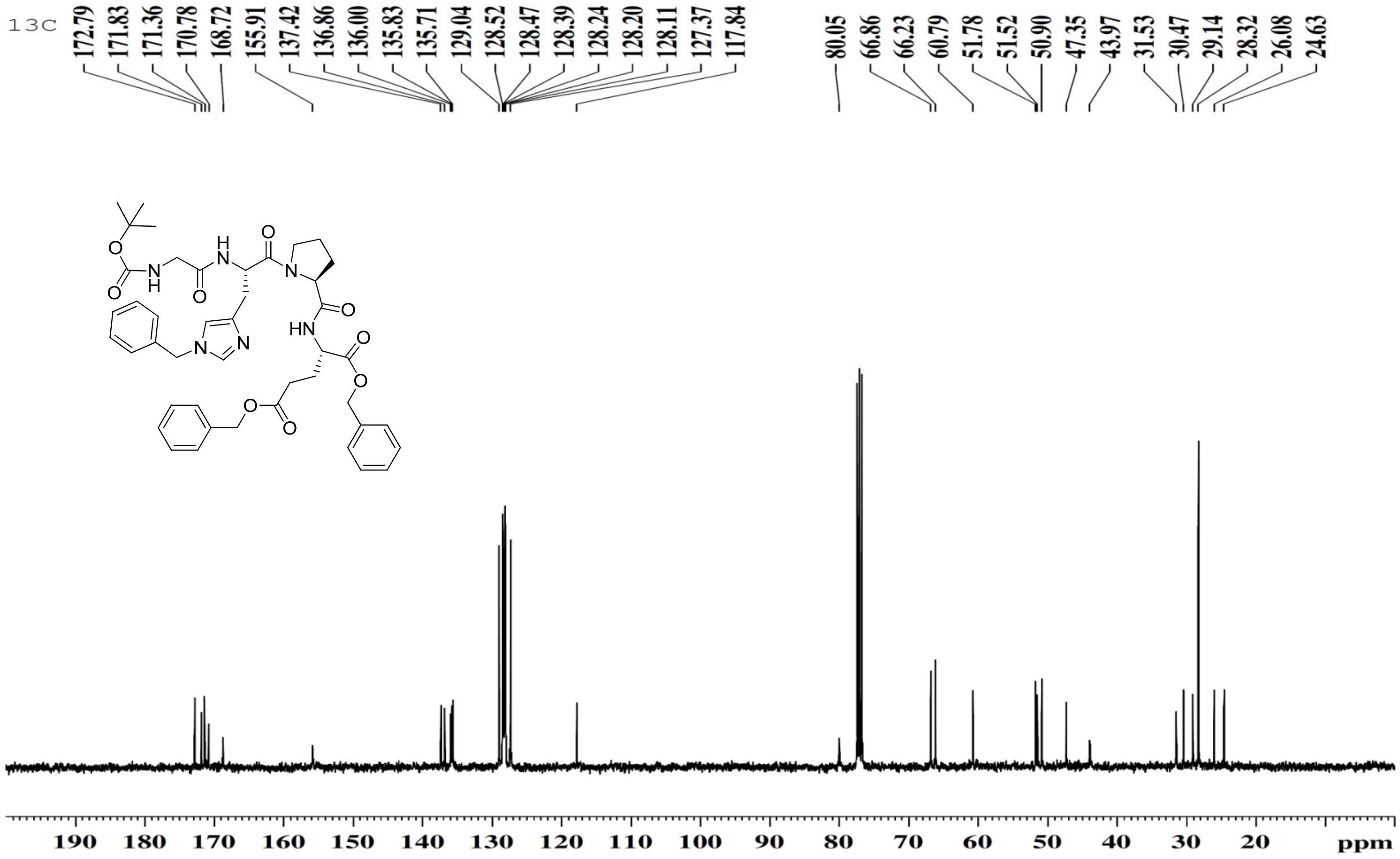

Figure S33. ^{13}C NMR: ($CDCl_3$, 100 MHz) of Boc-glycyl-L-histidinyl(OBn)-L-prolyl-L-glutamyl dibenzyl ester (compound **13**).

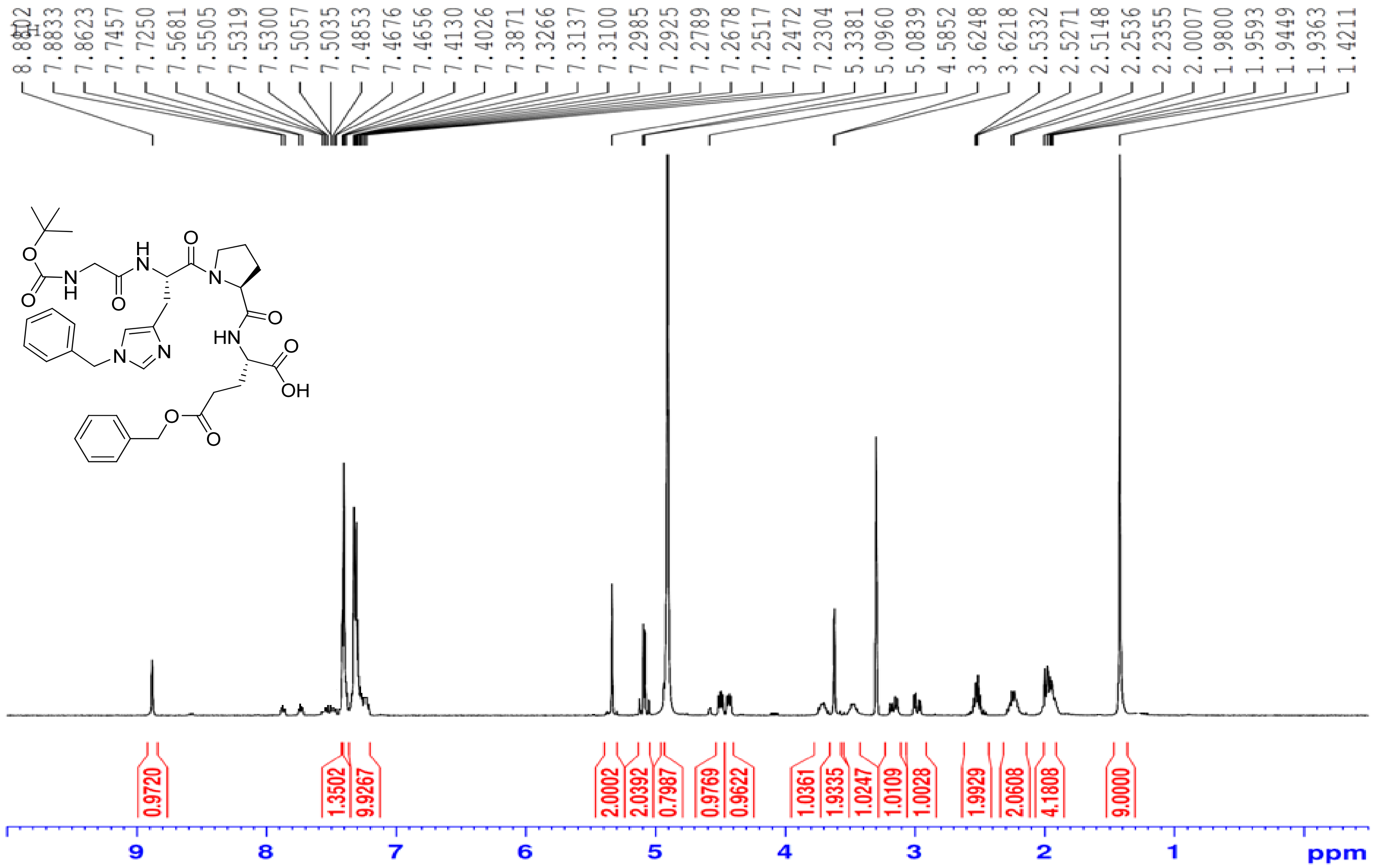

Figure S34. ^{1}H NMR: ($CDCl_3$,400 MHz) Boc-glycyl-L-histidinyl(OBn)-L-prolyl-L-glutamic acid-γ(OBn) (compound **14**).

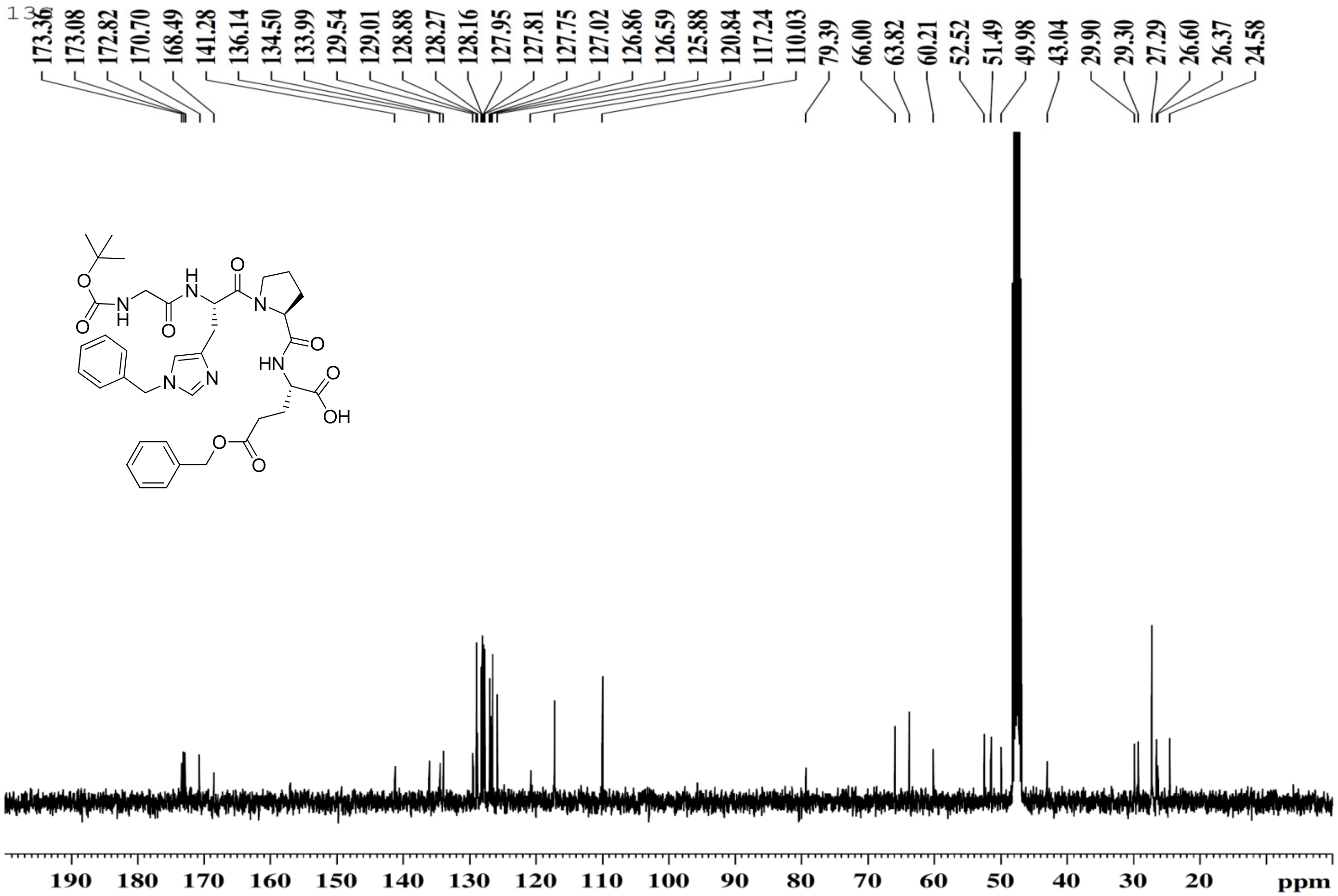

Figure S35. ^{13}C NMR: ($CDCl_3$,100MHz) Boc-glycyl-L-histidinyl(OBn)-L-prolyl-L-glutamic acid-γ(OBn) (compound **14**).

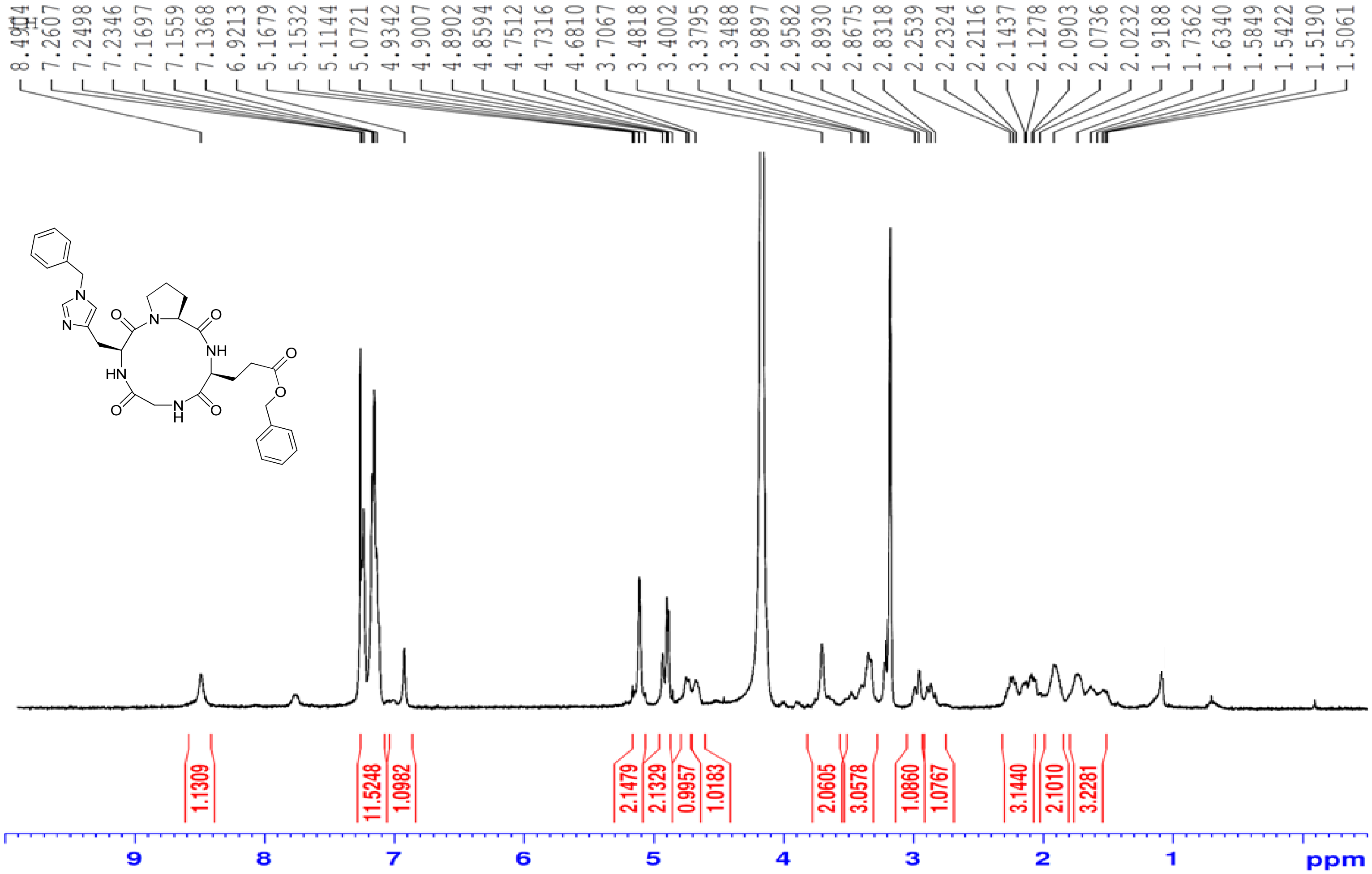

Figure S36. ^{1}H NMR: ($CDCl_3$ & CD_3OD,400 MHz) *Cyclo*(glycyl-L-histidinyl(OBn)-L-prolyl-L-glutamyl(OBn) (compound **4**).

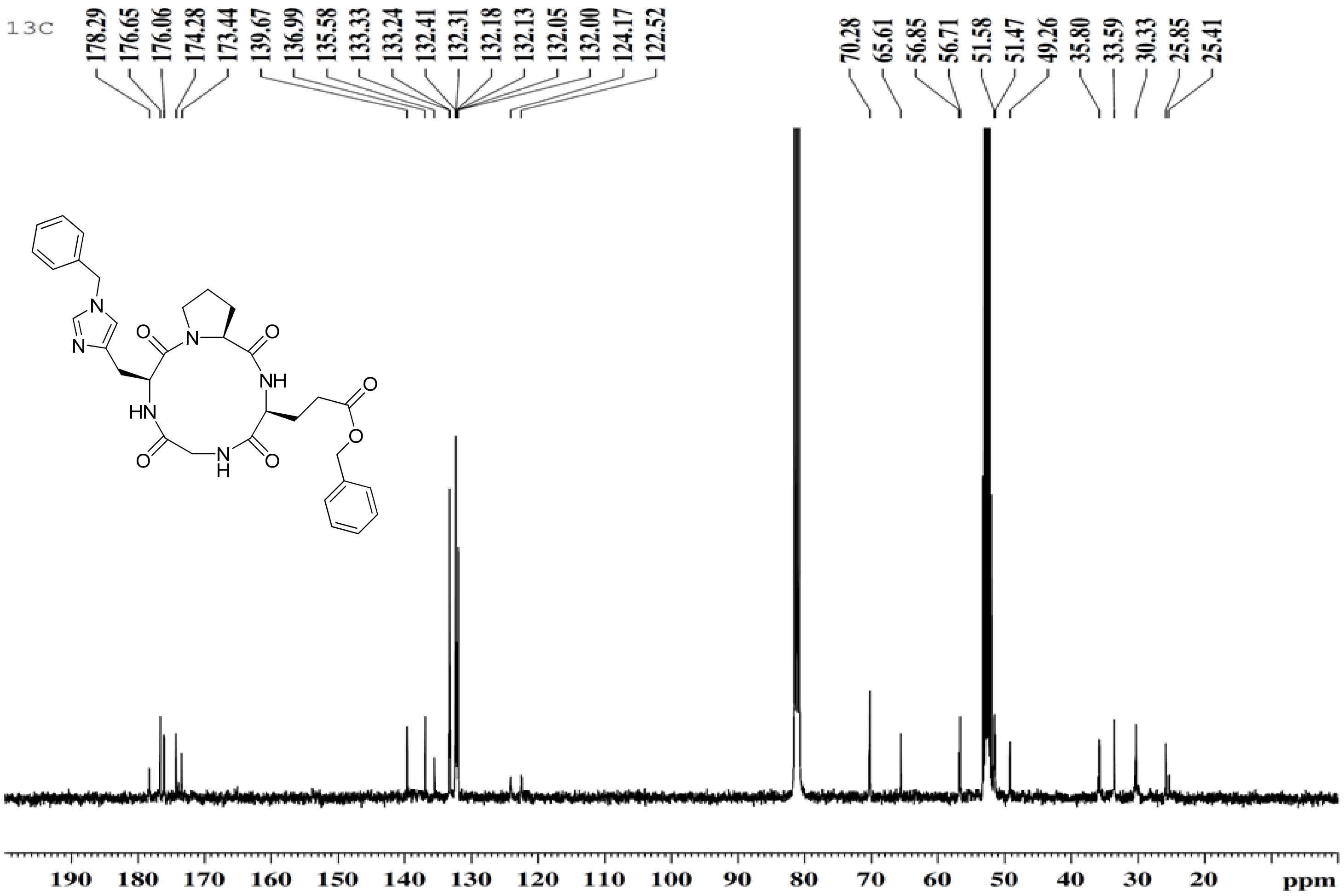

Figure S37. ^{13}C NMR: ($CDCl_3$ & CD_3OD,400 MHz) *Cyclo*(glycyl-L-histidinyl(OBn)-L-prolyl-L-glutamyl(OBn) (compound **4**).

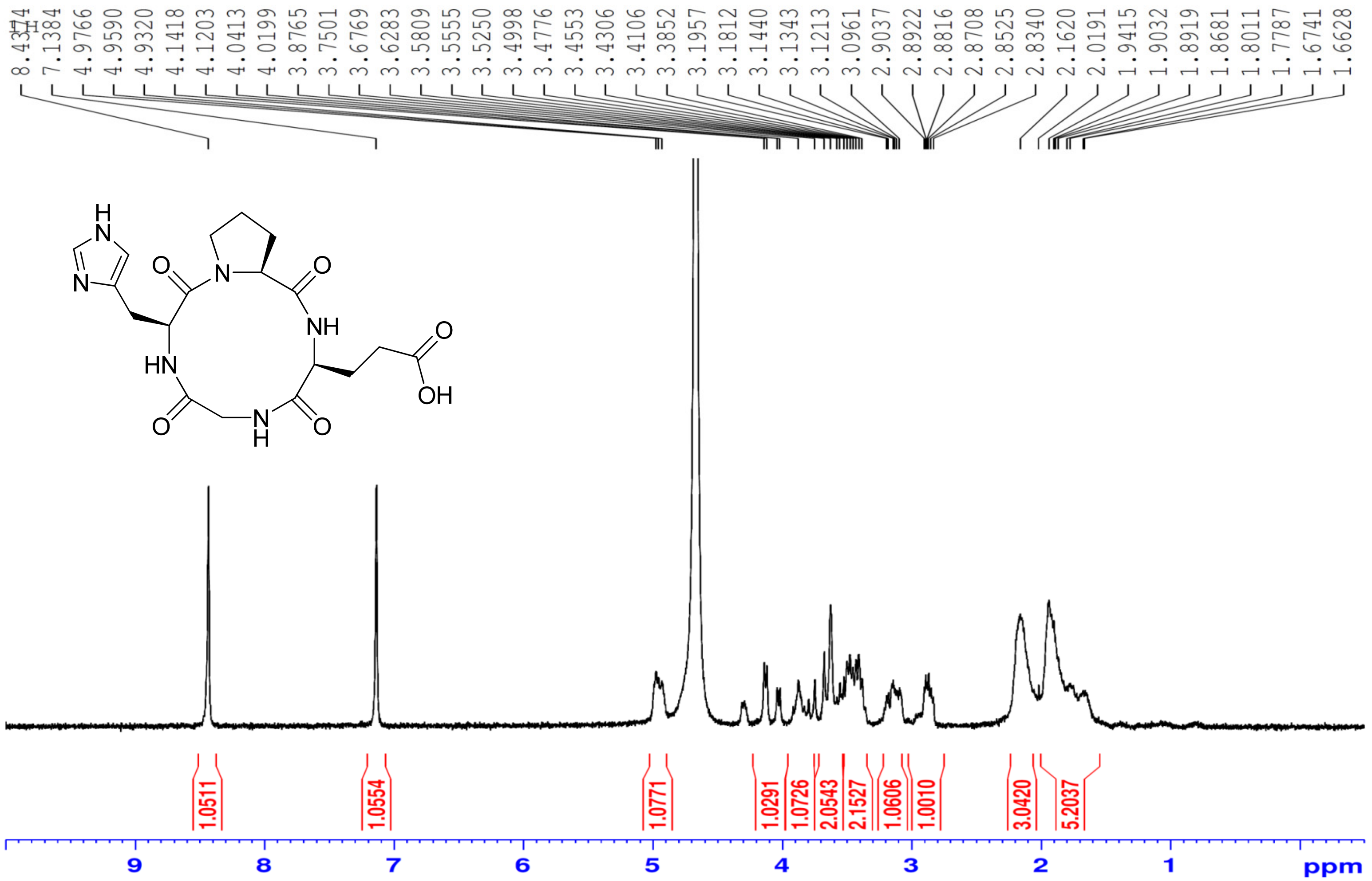

Figure S38. ^{1}H NMR: (D_2O, 400 MHz) *Cyclo*(glycyl-L-histidinyl-L-prolyl-L-glutamyl) (compound **2**).

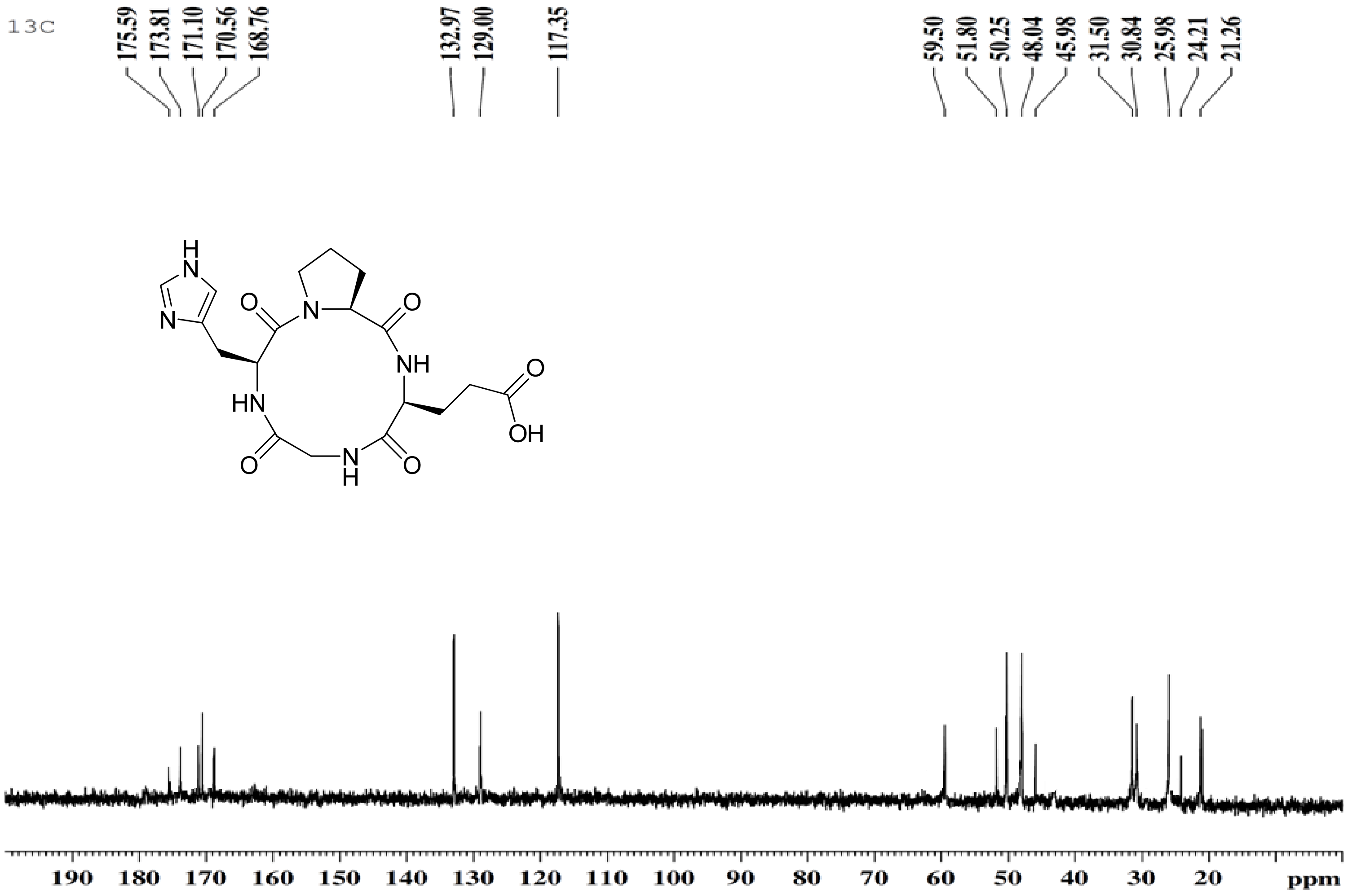

Figure S39. ^{13}C NMR: (D_2O, 400 MHz) *Cyclo*(glycyl-L-histidinyl-L-prolyl-L-glutamyl) (compound **2**).

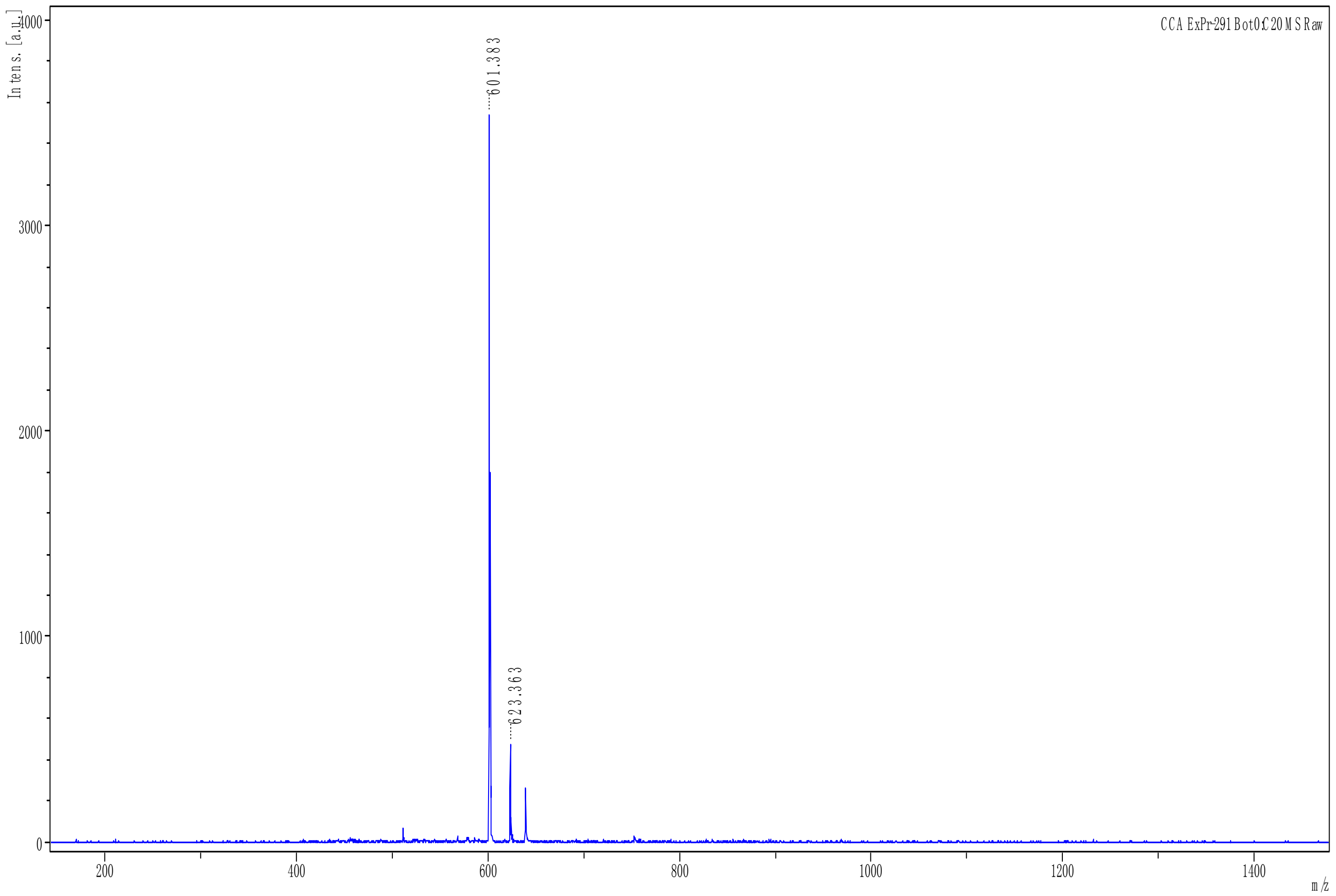

Figure S40. MALDI-TOF: *Cyclo*(glycyl-L-histidinyl(OBn)-L-prolyl-L-glutamyl(OBn) (compound **4**).

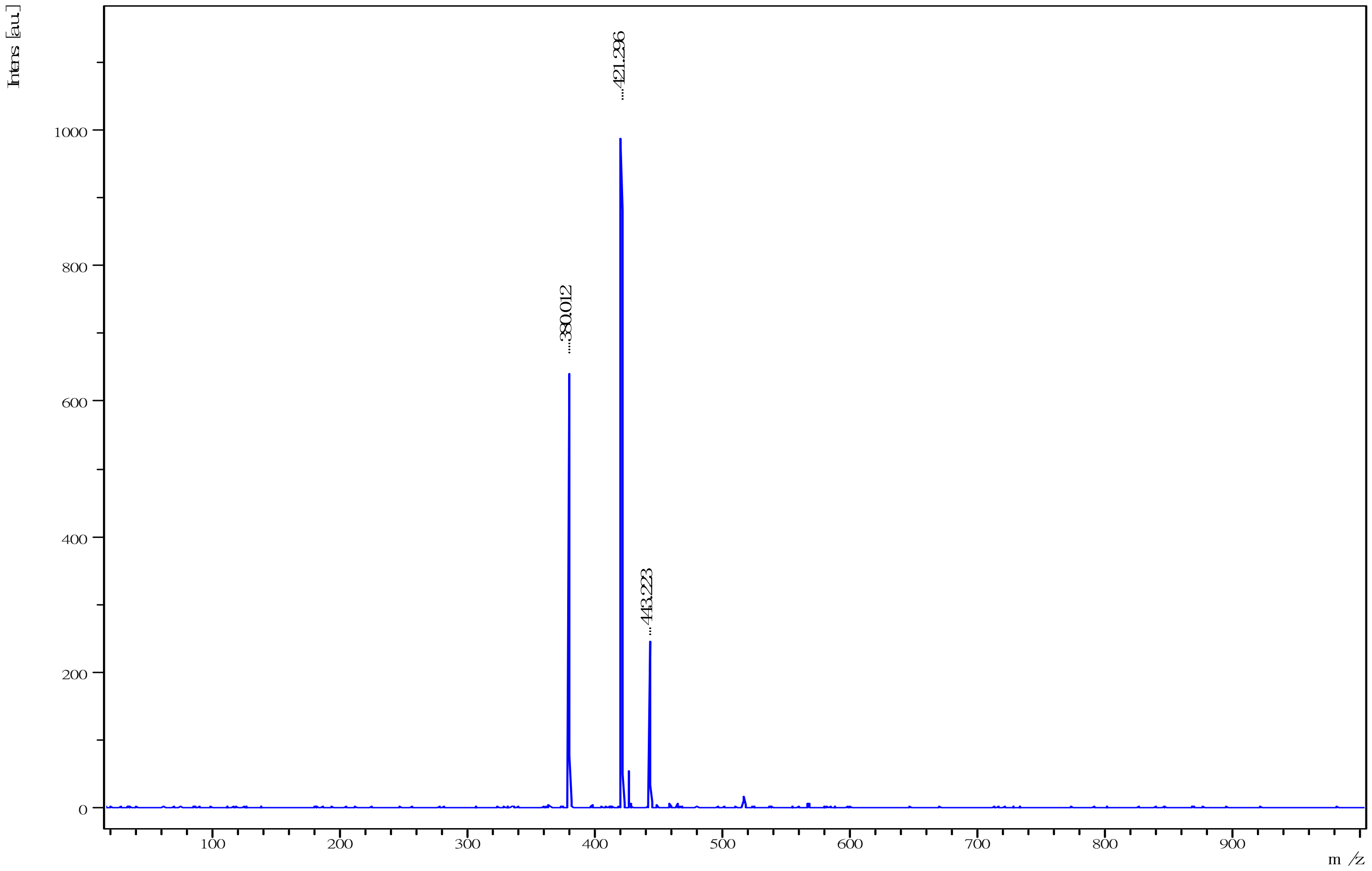

Figure S41. MALDI-TOF: *Cyclo*(glycyl-L-histidinyl-L-prolyl-L-glutamyl) (compound **2**).

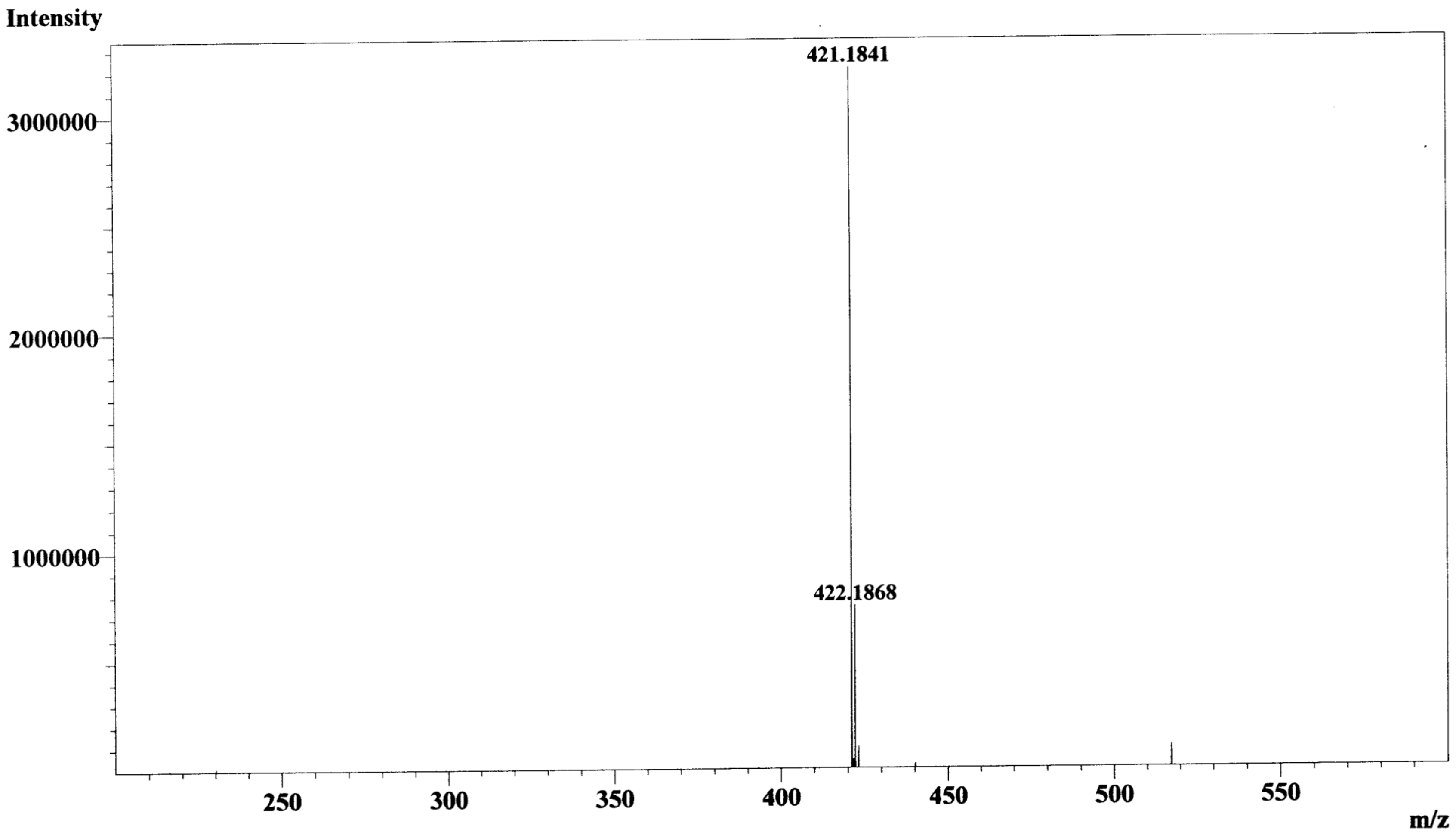

Figure S42. ESI-MS: *Cyclo*(glycyl-L-histidinyl-L-prolyl-L-glutamyl) (compound **2**).

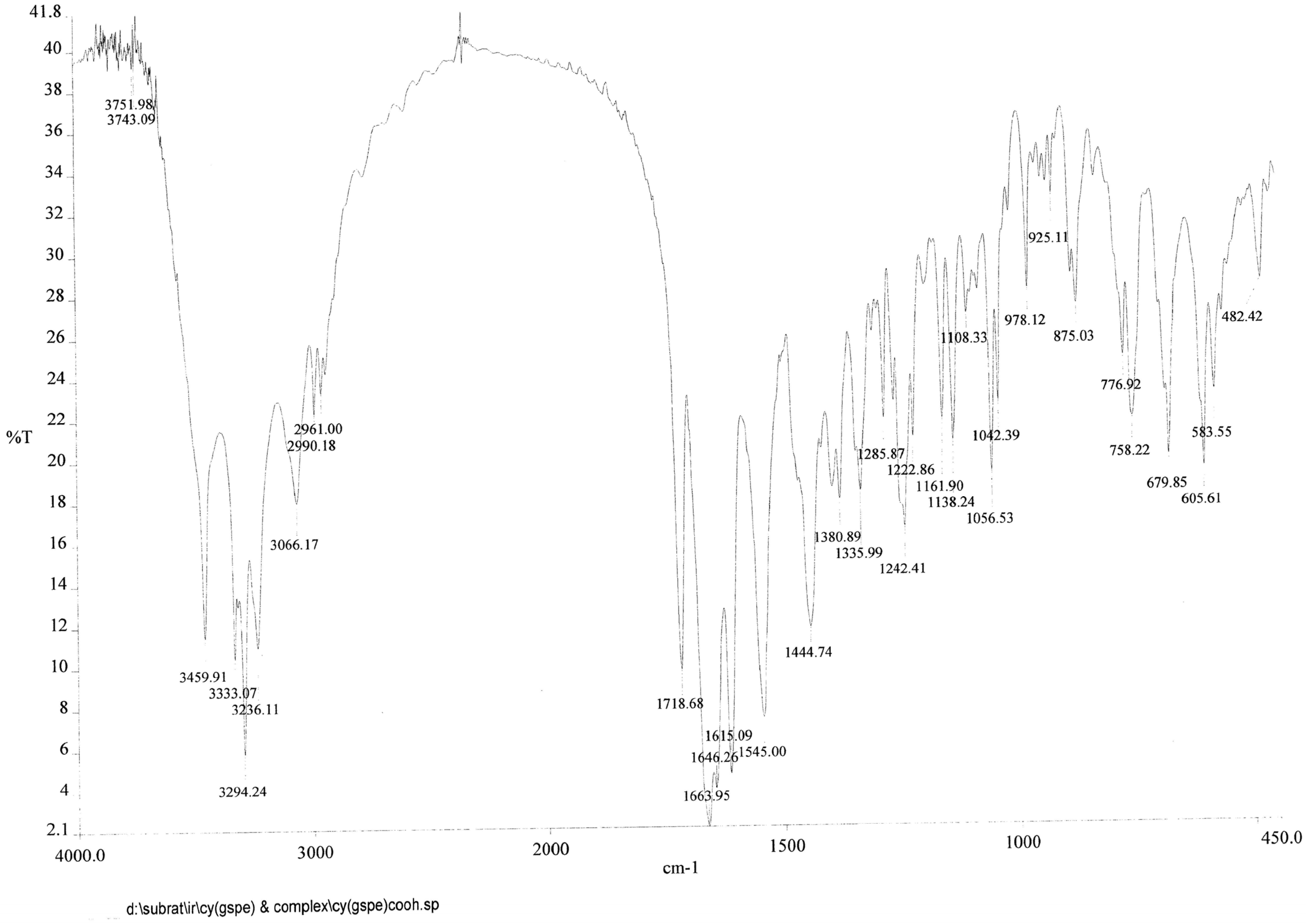

Figure S43. IR spectrum of *Cyclo*(glycyl-L-seryl-L-prolyl-L-glutamyl) (compound **1**).

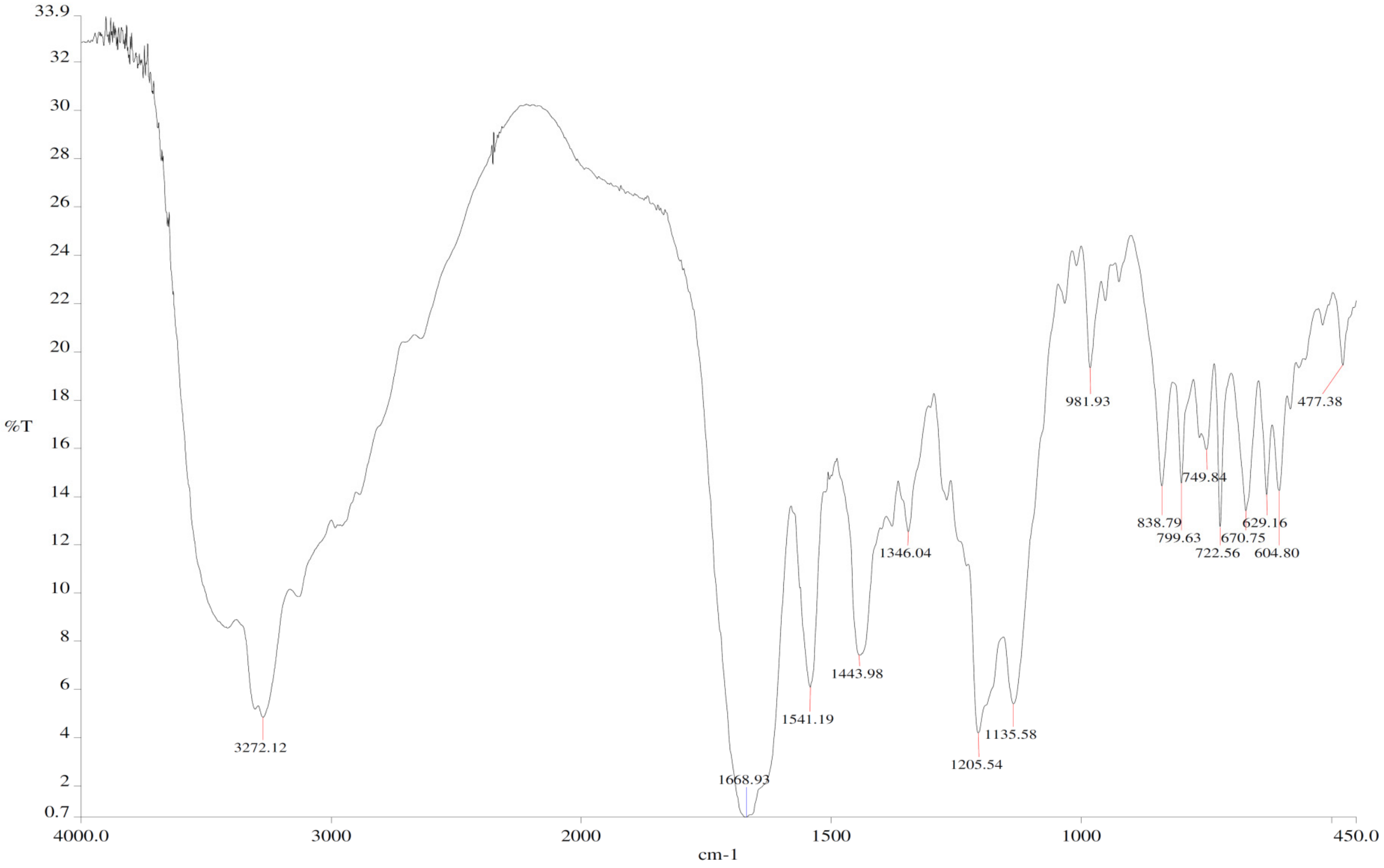

Figure S44. IR spectrum of *Cyclo*(glycyl-L-histidinyl-L-prolyl-L-glutamyl) (compound **2**).

© 2015 by the authors; licensee MDPI, Basel, Switzerland. This article is an open access article distributed under the terms and conditions of the Creative Commons Attribution license (http://creativecommons.org/licenses/by/4.0/).
